# Gas Sensing With Aerogels: A Critical Review on Structure‐Property Correlations and Performance Optimization

**DOI:** 10.1002/advs.76793

**Published:** 2026-07-27

**Authors:** Hao Guo, Linheng He, Qinxin Wang, Man Yuan, Shoutian Qiu, Wei Liu, Sheng Cui

**Affiliations:** ^1^ State Key Laboratory of Materials‐Oriented Chemical Engineering College of Materials Science and Engineering Nanjing Tech University Nanjing China; ^2^ Jiangsu Collaborative Innovation Center for Advanced Inorganic Function Composites Nanjing Tech University Nanjing China

**Keywords:** aerogel, composite materials, gas sensing, sensing mechanism, structure–property relationships

## Abstract

Gas sensing technology has significant applications in environmental monitoring, industrial safety, and disease diagnosis. Aerogel has become a key material for building high‐performance gas sensors through engineering design such as heterostructure construction and component compositing, with its high specific surface area, three‐dimensional interconnection network structure and rich interface characteristics. Nevertheless, the intrinsic structure property correlation of control aerogel gas sensors has not been systematically evaluated, and the key review of current performance optimization strategies remain inadequate. This paper systematically reviews the research progress of various aerogels in the field of gas sensing, with a focus on sensing aerogels including metal oxide semiconductors, sulfide semiconductors, and carbon‑based materials, as well as their sensing mechanisms. We systematically benchmark their sensing performance toward target analytes, such as nitrogen oxides, ammonia gas, and aldehydes. Subsequently, we systematically decipher the inherent links between key structural parameters (specific surface area, pore size distribution etc.) and core sensing metrics (sensitivity, selectivity, and response/recovery kinetics). Finally, this paper looks ahead to key challenges including establishing quantitative structure‐property relationships, improving environmental stability, engineering selectivity, and scaling up manufacturing, aiming to provide a unified research framework for the rational design and practical application of high‑performance gas‑sensitive aerogels.

## Graphical abstract

With high specific surface area, and adjustable interface, aerogel provides a multi‐functional platform for gas sensing. This review systematically links structural parameters (specific surface area, pore size, heterojunctions) to sensing metrics (sensitivity, selectivity, stability), critically evaluates optimization strategies, and outlines future directions toward rational design and practical deployment of high‐performance aerogel‐based gas sensors.

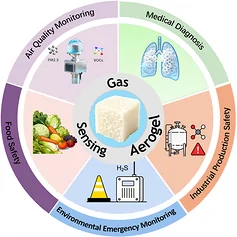

## 1 Introduction

**FIGURE 1:**
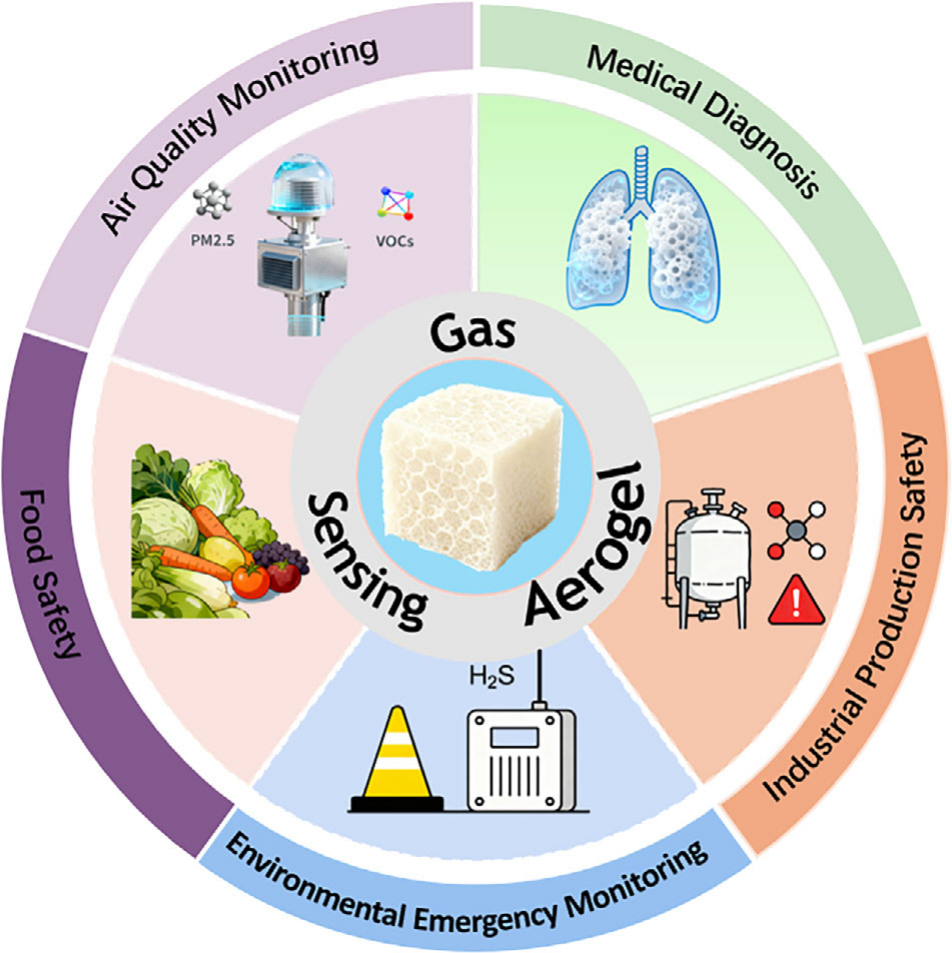
Applications of aerogel in different gas sensing.

Serve as pivotal mediators within Earth's ecosystems and biological processes, the composition and concentration of gases directly reflect environmental safety, productive activities, and human health [1, 2, 3]. For instance, NO_x_ exacerbates environmental issues such as acid rain and the greenhouse effect. Furthermore, trace gases exhaled by humans, including NH_3_ and acetone, may serve as potential biomarkers for disease diagnosis [4, 5, 6, 7, 8, 9]. Consequently, the precise identification, capture, and sensing of these specific gases have become core tasks in environmental monitoring, holding significant implications for advancing environmental governance, public safety, and biomedical fields (Figure 1) [10].

In recent decades, various gas sensing methods such as chromatography, spectroscopy, and fluorescence techniques have been explored [11, 12, 13]. However, although these methods are highly accurate, the necessary equipment is bulky and expensive, and requires professional operation, which limits their application in on‐site and distributed sensing [14, 15]. In contrast, resistive gas sensing technology demonstrates great potential in gas sensing in complex environments due to its unique advantages, including portable equipment, rapid response time, low cost, and real‐time electrical signal output [16, 17]. Nevertheless, significant challenges remain in achieving an ideal balance between sensitivity, selectivity, response speed, and cost. This directly restricts its application in real‐time monitoring, portable devices, and complex environments [18]. Furthermore, the performance of resistive sensing technology is also limited by the inherent defects of traditional electrode materials (such as nanoparticles and nanowires) [19, 20]. Such materials are highly prone to agglomeration during device fabrication, forming densely packed film structures. This buries many active sites and ultimately results in the sensing material having insufficient sensitivity and a slow response speed. The detection limit often remains at the ppm level, making it challenging to fulfill the urgent need for high‐precision sensing of low‐concentration gases [21, 22].

Aerogel is a porous material typically formed by the aggregation of nanoscale particles or polymer molecular chains [23]. Thanks to its core structural characteristics, such as low density, a high specific surface area, high porosity, and a large pore volume, this type of material has a wide range of potential applications in fields such as adsorption, catalysis, and sensing [24, 25, 26]. In the field of gas sensing, its structural advantages address the core challenges of traditional nanomaterials by maximizing the exposure of active sites and shortening gas diffusion pathways, thereby significantly enhancing the sensitivity and response speed of sensing materials. Concurrently, the highly designable surface and framework of aerogels permit the introduction of specific functional groups (such as –NH_2_, –OH) or active components (e.g., noble metal nanoparticles) through doping or modification techniques. This enables precise regulation of selective sensing and reactivity toward target gases, effectively enhancing the sensing material's resistance to interference and long‐term stability [27, 28, 29]. Furthermore, specific interactions, such as hydrogen bonding and electrostatic adsorption, between the aerogel skeleton and gas molecules offer new possibilities for improving sensing selectivity further [30].

Research on aerogels in the field of gas sensing primarily focuses on material innovation and performance optimization. However, the latest progress in the practical application of high‐performance, collaborative improvements to resistive aerogel gas sensing materials is still lacking in systematic integration and carding. Therefore, a comprehensive review of the latest research results on aerogels in gas sensing is highly significant. This article provides a systematic summary of the classification, design, and sensing mechanisms of aerogels in various gas detection applications, as well as their excellent performance. First, the core sensing mechanisms of aerogels under high‐temperature and normal‐temperature conditions are explained in detail. Second, the structural functionalization methods of aerogel materials are summarized, including heterostructure construction and doping modification, with the aim of obtaining sensing materials with high sensitivity and selectivity. Third, the use of aerogels in detecting gases such as nitrogen oxides, ammonia, and aldehydes is explored. Finally, the current technical challenges and future development directions are discussed.

## 2 Classification and Design of Aerogels

### 2.1 Metal Oxide Semiconductor Aerogel

In the field of gas sensing materials research, metal oxide semiconductors have long been the preferred choice for sensing elements due to their core advantages of high chemical stability, straightforward preparation processes, and ease of miniaturization [31, 32, 33]. Aerogels, with their ultra‐high specific surface area and 3D interconnected nanonetwork structure, can further increase the exposure of active sites in metal oxides and shorten gas diffusion pathways, providing crucial structural support for enhancing sensing performance [34]. Early investigations into such aerogels primarily focused on optimizing synthesis processes and regulating microstructures. Presently, aerogel systems based on CuO, SnO_2_, and TiO_2_ have emerged as research hotspots due to their concurrent high sensitivity and selectivity in specific gas sensing applications. This trend is accelerating the advancement of metal oxide semiconductor aerogels toward high‐performance sensing applications [35, 36].

#### 2.1.1 Copper Oxide‐Based Composite Aerogel

CuO is a p‐type metal oxide characterized by a narrow bandgap of approximately 1.2 eV, plentiful raw materials, straightforward preparation, low toxicity, and excellent room‐temperature gas sensitivity. Consequently, it has been widely studied for sensing various gases [37]. For instance, Cheng et al. [38] developed rGO‐CuO/WO_3_ nanocomposite materials that exhibit high selectivity for acetone gas at an optimal operating temperature of 320°C and possess good repeatability and long‐term stability. Zhu et al. [39] prepared oxygen‐rich vacancy porous spherical CuO with oxygen‐rich vacancies using a thermal solvent reduction method. This material showed the best response to ammonia gas at 200°C, however, as the temperature increased, the resistance also increased, resulting in decreased selectivity for ammonia. Indeed, as the operating temperature of traditional CuO‐based sensing materials increases, so does the resistance, resulting in reduced gas selectivity. While strategies such as composite modification and microstructure regulation can improve the overall performance of sensing materials within a specific temperature range, these shortcomings remain unresolved. This technical bottleneck limits the promotion and application of CuO‐based gas sensing materials in portable devices and poses potential safety hazards in flammable and explosive gas sensing scenarios.

**FIGURE 2:**
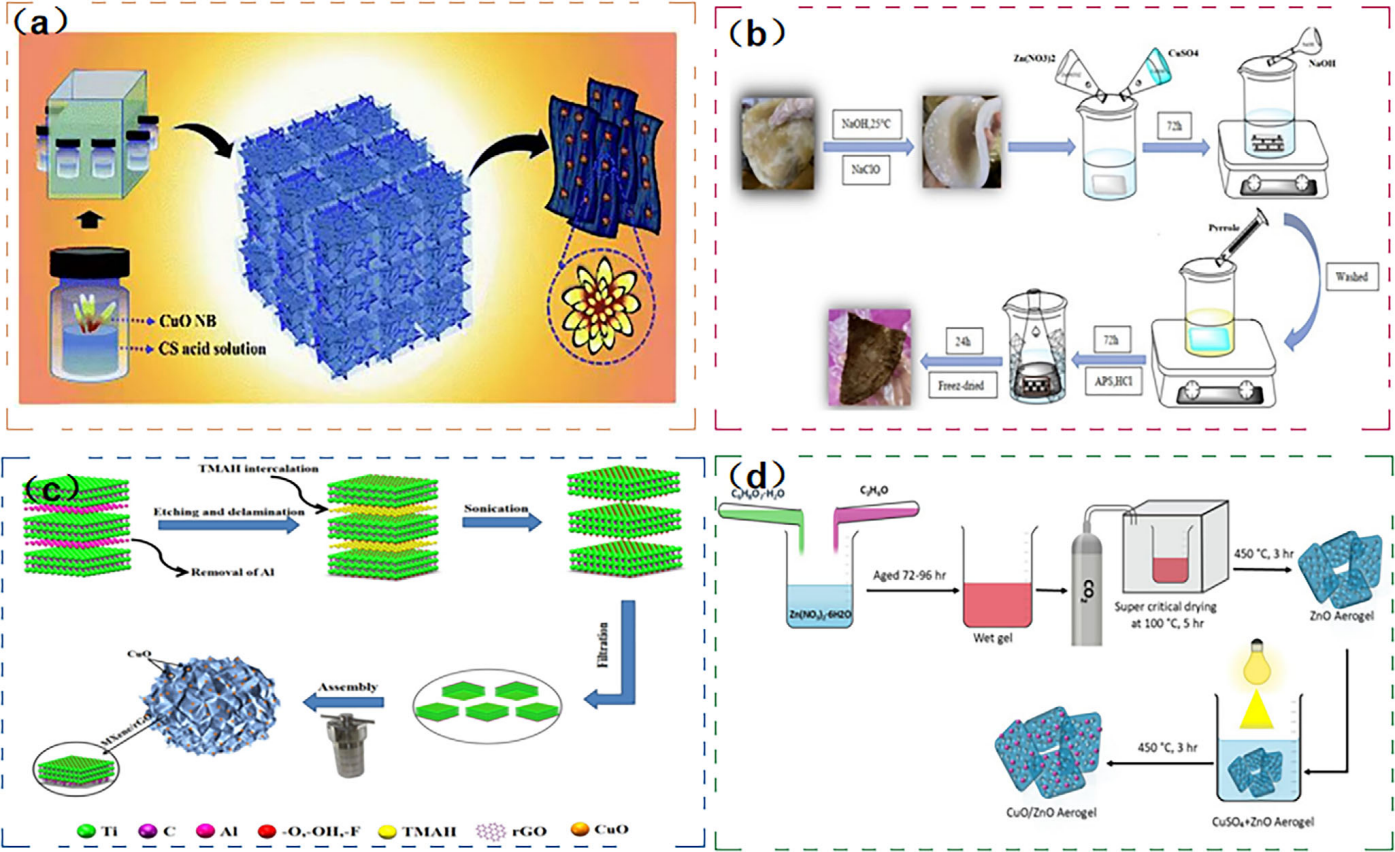
(a) Schematic diagram of the preparation of Cu/Cu_2_O/CuO carbon aerogel. Reproduced with permission [42]. Copyright 2021, The Royal Society of Chemistry. (b) Schematic diagram of the synthesis of BC/CuO/Zn/Py aerogel, emphasizing NaOH treatment, NaOCl bleaching, and pyrrole polymerization. Reproduced with permission [44]. Copyright 2025, Elsevier. (c) Schematic diagram of the preparation process of 3D MXene/rGO/CuO aerogel. Reproduced with permission [45]. Copyright 2021, Elsevier. (d) Schematic diagram showing the preparation of CuO/ZnO aerogel via an epoxide‐initiated gelation process. Reproduced with permission [46]. Copyright 2025, Elsevier.

To address the above hidden dangers, researchers have turned their attention to aerogel materials, which have a 3D porous network structure. Compared to ordinary composite materials or thin films, aerogels have a huge specific surface area and high porosity. Their interconnected pore structures provide ideal pathways for the bulk diffusion and rapid transport of gas molecules, while also facilitating the full exposure of active sites. This lays the foundation for reducing the operating temperature of sensing materials by a significant amount [40, 41]. The construction of CuO‐based composite aerogels can significantly enhance the sensitivity and response speed of sensing materials while significantly reducing the operating temperature. Jing et al. [42] synthesized Cu/Cu_2_O/CuO@CA aerogels (Figure 2a) using a freeze‐drying‐calcination strategy. This aerogel has a specific surface area of this aerogel is 42.81 m^2^/g, accelerating the mass transfer process during the reaction. The CuO component enhances the aerogel's chemical stability and disperses uniformly throughout its structure in composite form, preventing the agglomeration of active components and maximizing the exposure of active sites. At the same time, the porous structure of the aerogel exhibits good conductivity, enhancing electron transport capability. Liu et al. [43] constructed a K_2_O‐Cu/carbon aerogel (CA) that leveraged the material's immense specific surface area (227.8 m^2^·g^−^^1^) and the rapid gas diffusion pathways and abundant adsorption sites provided by its 3D mesoporous structure. This enabled the efficient dispersion and immobilization of CuO nanoparticles, fundamentally preventing particle migration, agglomeration, and sintering. Nastaran et al. [44] prepared bacterial cellulose (BC)/PPy/CuO/ZnO aerogels (Figure 2b) using the freeze‐drying method. After adding CuO, the material's conductivity increased from 10^−8^∼10^−7^ to 2.2 × 10^−2^ S/cm, and its degradation temperature also increased from 280°C to 315°C, significantly enhancing its conductivity and thermal stability. The large specific surface area of the aerogel provides an ideal site for gas reactions. Liu [45] constructed a 3D aerogel skeleton using MXene and rGO nanosheets, and uniformly distributed CuO nanoparticles on the network surface. This process successfully preparing MXene/rGO/CuO composite aerogels (Figure 2c). The 3D interconnected porous structure of the aerogel greatly facilitates the diffusion and adsorption of acetone gas. This enables the sensing material to achieve a high response of 52.09% to 100 ppm of acetone at room temperature, while exhibiting extremely fast response and recovery times of ∼6.5 and ∼7.5 s, respectively.

In addition to optimizing the 3D macrostructure, constructing heterojunctions in aerogels is a key strategy for enhancing their performance further. Jaihindh et al. [46] successfully loaded CuO nanoparticles onto the surface of ZnO aerogels by combining an epoxide‐induced gelation process with photo deposition technology, thereby forming a CuO/ZnO heterojunction (Figure 2d). This formed heterojunction interface can effectively promote charge separation, significantly enhance the material's redox capability of the material and provide an efficient pathway for regulating its electronic structure. This structural design also increases the material's specific surface area (27.91 m^2^/g) and porosity, optimizing its sensing performance through the synergistic effect of multiple factors. Xu et al. [47] prepared Cu@CuO in an aerogel. In this material, CuO is compounded with metallic copper to form a Cu@CuO heterojunction. The high conductivity of metallic copper significantly reduces the material's overall resistance, while the semiconductor properties of CuO ensure a sensitive gas‐sensing response. The conductivity and catalytic stability of the material are improved under the synergistic effect of the two, providing a reliable basis for the stable output of electrochemical signals. Concurrently, the aerogel's 3D open porous interconnected structure, characterized by its ultra‐low density and high specific surface area, further facilitates mass transfer processes for reactants and enhances electron transport efficiency.

#### 2.1.2 Tin Oxide‐Based Composite Aerogel

SnO_2_, as a typical n‐type metal‐oxide‐semiconductor (MOS) gas sensing material, operates based on the interaction between gas molecules and the material's surface [48, 49]. However, traditional SnO_2_ sensing materials have exhibited significant limitations. Their limited specific surface area results in insufficient exposure and utilization of active sites, while their dense structure hinders gas mass transfer, slowing the process of gas adsorption and desorption. These defects directly limit their gas sensing performance, failing to meet the demand for high‐sensitivity detection [50, 51, 52]. To improve the gas sensing performance of traditional SnO_2_, researchers have attempted to modify it by targeting low‐dimensional structures [53]. For example, Liu et al. [54] significantly increased the sensitivity of SnO_2_ thin films to H_2_S at 100°C by doping with copper, while Ajay K. Sao [55] developed a SnO_2_/CdS hybrid material that could rapidly detect 50 ppb trace NO_2_ at 90°C. Ema et al. [56], meanwhile, constructed a CeO_2_/SnO_2_ nanosheet heterojunction that utilizes interface barriers to modulate the width of the electron depletion layer and amplify the resistance change signal after the material contact with gas. Nevertheless, the material morphologies in these modification efforts are mostly low‐dimensional stacked structures, which still suffer from inherent problems such as insufficient adsorption capacity and limited mass transfer efficiency, making it difficult to achieve a breakthrough in performance [57].

**FIGURE 3:**
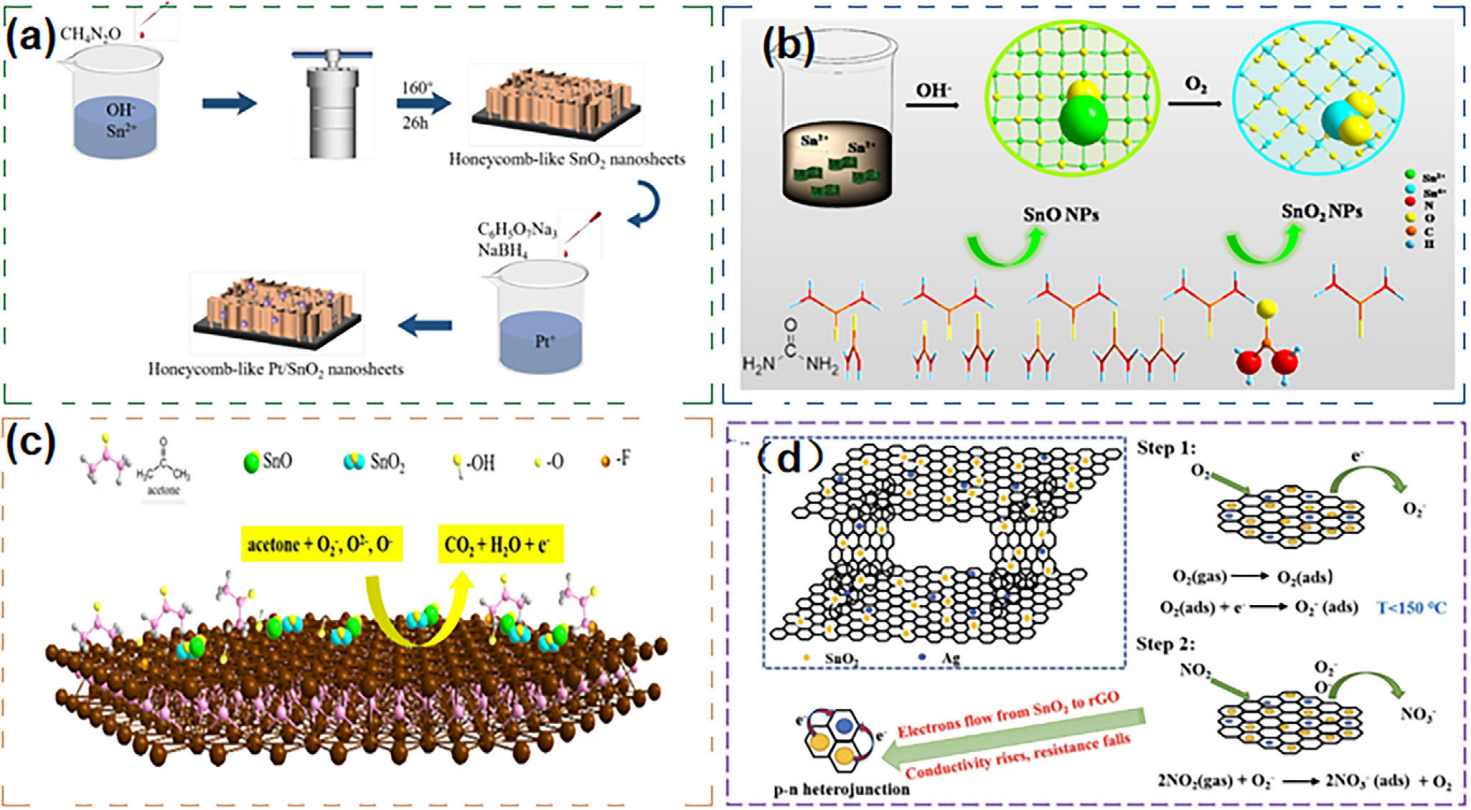
(a) Schematic diagram of the preparation process of honeycomb‐like Pt/SnO_2_ nanosheets. Reproduced with permission [58]. Copyright 2023, Elsevier. (b) Schematic diagram of the chemical reactions during the preparation of SnO‐SnO_2_/Ti_3_C_2_T_x_ nanocomposite materials. Reproduced with permission [60]. Copyright 2021, Elsevier. (c) Schematic diagram of the band structure of the "hamburger‐like" p‐n heterojunction. Reproduced with permission [60]. Copyright 2021, Elsevier. (d) Gas sensitivity mechanism of SnO_2_/rGO aerogel. Reproduced with permission [61]. Copyright 2021, Elsevier.

To address these issues, researchers have further explored the optimization of the performance of SnO_2_‐based aerogels. For instance, Sun et al. [58] synthesized Pt‐SnO_2_ composite aerogels that exhibited an impressive response value of 3700 (R_a_/R_ɡ_) to 100 ppm triethylamine at 200°C, clearly demonstrating the synergistic impact of noble metal modification and aerogel's structure (Figure 3a). Liu et al. [59] constructed a 3DMXene/rGO/SnO_2_ composite aerogel via a one‐step hydrothermal method, enabling in situ growth of SnO_2_ nanoparticles on the MXene/rGO two‐dimensional heterointerface. whose particle size precisely matched the aerogel's mesoporous aperture (2.9 nm). This arrangement simultaneously filled interlayer voids to prevent MXene stacking while preserving the through‐pore structure, elevating the specific surface area to 103.2 m^2^/g. The aerogel's exceptionally large specific surface area provides ample pathways and sites for formaldehyde adsorption and mass transfer. Crucially, the incorporation of SnO_2_ also enhances the structural stability of the aerogel, addressing the issues of pure MXene's tendency to stack and rGO's insufficient mechanical properties.

The core components for constructing p‐n heterojunctions are also pivotal for enhancing gas‐sensing response and selectivity at room temperature. Wang et al. [60] prepared an SnO‐SnO_2_/Mxene composite aerogel, in which some of the p‐type SnO was converted to n‐type SnO_2_ during the preparation process, thus forming an in situ SnO‐SnO_2_ p‐n heterojunction on the SnO surface (Figure 3b). This heterojunction acts as an active unit within the two‐dimensional layered MXene framework, forming a “hamburger‐like” composite structure (Figure 3c). The interconnected porous structure of the aerogel prevents the easy agglomeration of SnO_2_, and the material's large specific surface area (46.7 m^2^/g) provides sufficient active sites for gas adsorption, thereby enhancing the gas sensing response to acetone at room temperature. Yan et al. [61] prepared an Ag‐SnO_2_/rGO aerogel using a sol‐gel method and freeze‐drying technology. The SnO_2_ particles were tightly fixed to the rGO sheets to form a p‐n heterojunction. The material exhibited pseudocapacitive characteristics, enabling the rapid storage and release of electrons and providing a new electron transport pathway to enhance the gas sensing signal response (Figure 3d). Yan et al. [62] also prepared an acidified SnO_2_/rGO aerogel. Many defect sites were formed on the SnO_2_ surface through etching with concentrated hydrochloric acid, increasing the material's specific surface area from 68.7 m^2^/g to 94.6 m^2^/g and enhancing the active sites. Subsequently, the acid‐etched SnO_2_ was compounded with rGO to form a 3D porous aerogel with a p‐n heterojunction. This aerogel's microporous structure (average pore diameter of 3.2 nm) and chemical adsorption characteristics of this aerogel enable it to detect ethanol down to a limit of 0.25 ppm, with a response value of 137.4 at 20 ppm.

#### 2.1.3 Titanium Dioxide Aerogel

As a typical wide‐bandgap semiconductor, TiO_2_ possesses advantages such as strong oxidizability, high catalytic activity, chemical stability, non‐toxicity and low cost, making it an ideal intrinsic gas‐sensing material in the field of gas sensing [63, 64, 65, 66]. However, the use of traditional TiO_2_ materials is limited by factors such as a small specific surface area, low gas mass transfer efficiency, and insufficient utilization of active sites, which makes it difficult to achieve high sensitivity and a rapid response in terms of sensing performance [67, 68, 69]. Therefore, transforming it into a 3D porous aerogel becomes an effective improvement strategy. TiO_2_ aerogels exhibit high tunability in terms of component ratio and pore size. They also allow for the customized design of gas‐sensing materials according to the detection requirements of target gases. This significantly expands their application potential in the field of gas sensing [70, 71, 72]. Early research by Ding et al. [73] involved preparing composite aerogels by embedding TiO_2_ nanoparticles into a fiber skeleton through an electrospinning process to achieve a low‐density, low‐thermal‐conductivity material. However, the introduction of TiO_2_ led to an increase in fiber defects, resulting in a decrease in overall mechanical strength. Yu et al. [74] used a hydrothermal method to grow TiO_2_ nanolayers and nanorods on the fiber surface before composing aerogels. This improved the uniformity of TiO_2_ loading, although it enhances the uniformity of TiO_2_ loading, it suffers from the drawbacks of a cumbersome process and stringent growth conditions.

**FIGURE 4:**
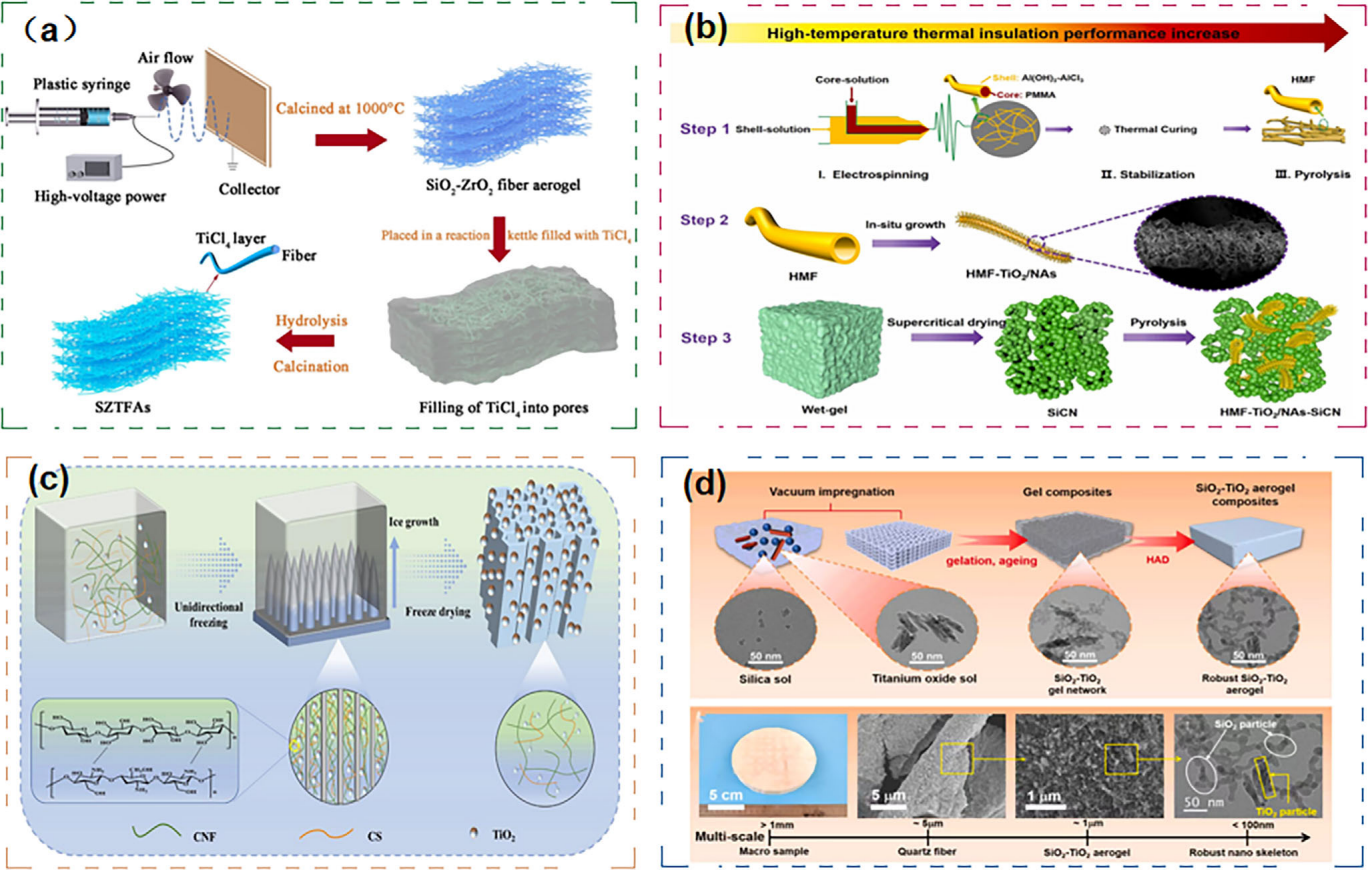
(a) Preparation scheme of SZTFA. Reproduced with permission [75]. Copyright 2025, Springer nature. (b) Preparation of HMF‐TiO_2_/NAs‐SiCN ceramic aerogel. Reproduced with permission [76]. Copyright 2023, Elsevier. (c) Preparation process of CNF/TiO_2_/CS aerogel. Reproduced with permission [77]. Copyright 2023, Elsevier. (d) Schematic diagram of the HAD process for STAC, as well as SEM and TEM images of multi‐scale composite materials at different scales. Reproduced with permission [78]. Copyright 2025, Elsevier.

In response to the above issues, Han et al. [75] developed TiO_2_ ceramic fiber composite aerogels (SZTFAs) (Figure 4a) via a synergistic process combining hydrothermal growth and surface coating to achieve uniform loading of TiO_2_ in the fiber aerogels. This material boasts ultra‐low density (14 mg/cm^3^), excellent mechanical properties (80% compression strain and 105.6 kPa compressive strength), and high‐temperature stability of up to 120°C. Wang et al. [76] prepared hollow mullite fiber structures via coaxial electrospinning and in situ growth of titanium dioxide nanorods on the fiber surface. Subsequently, by filling the pores of the TiO_2_ nanorod composite with SiCN aerogel successfully prepared a ternary composite ceramic aerogel material was successfully prepared (Figure 4b). The incorporation of titanium dioxide nanorods enhances the interfacial bonding between fibers and aerogels within the composite material (achieving a compressive strength of 392 kPa), without altering its density. These two straightforward preparation methods provide significant reference for TiO_2_ aerogels used in sensing applications, demonstrating that synergistic design of the fiber network and aerogel framework can ensure mechanical stability while achieving efficient exposure of TiO_2_ active sites.

In addition to the above composite strategies utilizing inorganic fibers as the reinforcement skeleton, researchers have also explored green synthesis pathways and novel drying techniques based on biomass templates. This has further expanded the structural design and preparation methods of TiO_2_ aerogels. Zhang et al. [77] cross‐linked two biomass materials, nanocellulose (CNF) and chitosan, using a cross‐linking agent to form a stable skeletal structure. They then anchored titanium dioxide nanoparticles onto the abundant amino and carboxyl groups on the chitosan surface. The resulting aerogel (CNF/TiO_2_/CS) has a 3D interconnected network structure was prepared using freeze‐drying (Figure 4c). This aerogel has a density of only 12.92 mg·cm^−3^ and possesses abundant active sites. Furthermore, it exhibits high stability. On the other hand, in terms of process innovation, Xie et al. [78] proposed a novel hydrothermal‐assisted drying process for preparing SiO_2_‐TiO_2_ aerogel composites. This hydrothermal‐assisted drying process is carried out in a controlled high‐temperature and high‐pressure hydrothermal environment to form a strong and tough 3D network structure (Figure 4d). This process provides a new way to make high‐performance aerogel composites.

Although the above researchers have optimized the preparation method, improvements to the gas sensing activity are still needed. The core strategy for enhancing the intrinsic gas sensing activity of TiO_2_ aerogels is defect engineering involving metal doping and crystal phase regulation. Metal atom doping introduces many oxygen vacancy defects into the TiO_2_ lattice. These defects act as adsorption centers that preferentially bind gas molecules, and they can also regulate the carrier concentration and transport efficiency of the material. This significantly amplifies the gas sensing response signal. For instance, Sui et al. [79] synthesized Pd‐doped TiO_2_ aerogels that utilize the electron‐induced effect of Pd atoms to generate high‐density oxygen vacancies. This greatly promotes the dissociative adsorption of hydrogen molecules and interfacial charge transfer, achieving improved sensing performance. Precise regulation of the crystal phase is crucial to ensure the effectiveness of the defects. Chen et al. [80] synthesized pure phase anatase TiO_2_ aerogels, which exhibit excellent comprehensive performance at 450°C for 100 ppm hydrogen, with a sensitivity of 3.1271 and a response/recovery time of only 4 s/29 s. Moreover, they demonstrate a good linear response in the concentration range of 100–1000 ppm, as well as high selectivity toward interfering gases such as ammonia and methanol. These performance advantages stem from the aerogel's porous structure, which enables rapid mass transfer, and the strong adsorption activity of the oxygen defects inherent in the pure phase anatase lattice.

Despite significant improvements in sensor performance through existing research, the issue of high energy consumption remains prominent. Consequently, constructing heterointerfaces to simultaneously optimize electronic structure and mass transfer efficiency has emerged as a key approach to reducing operational power consumption. Ramos et al. [81] employed the sol‐gel method to fabricate TiO_2_carbon gel composite aerogels, establishing a 3D interconnected conductive network. The composite system of TiO_2_with two‐dimensional materials such as graphene can accelerate charge transport through the high conductivity of the two‐dimensional materials. Simultaneously, the heterojunction formed between the two materials can effectively regulate the carrier separation efficiency, achieving a synergistic enhancement in both the sensitivity and response speed of the sensing material.

### 2.2 Sulfide Semiconductor Aerogel

As an emerging material in the field of gas sensing, sulfide semiconductor aerogels have tremendous application potential thanks to their unique surface chemical properties and precisely controllable electronic structure [82, 83]. Unlike traditional metal oxide gas sensing materials, sulfides such as molybdenum disulfide (MoS_2_) and cadmium sulfide (CdS) have surfaces rich in sulfur vacancies. These can serve as highly selective active sites capable of preferential adsorption and directed reaction toward specific gas molecules, such as nitric oxide (NO_2_) and ammonia (NH_3_) [84, 85].

#### 2.2.1 Molybdenum Disulfide Aerogel

Of the many sulfides, molybdenum disulfide (MoS_2_), with its two‐dimensional layered structure, has attracted significant attention in the field of gas sensing thanks to its suitable bandgap, high carrier mobility, and high catalytic activity at edge sites [86, 87]. However, the van der Waals forces inherent in two‐dimensional nanosheets tend to cause severe stacking, burying many highly active edge sites and thus significantly restricting their full gas sensing performance [88]. The key to solving this problem is assembling two‐dimensional MoS_2_ nanosheets into a 3D aerogel. The interconnected porous 3D network of the aerogel effectively inhibits the re‐stacking of nanosheets to form an open, permeable structure that fully exposes their highly active edge sites and provides ideal channels for the rapid diffusion of gas molecules.

**FIGURE 5:**
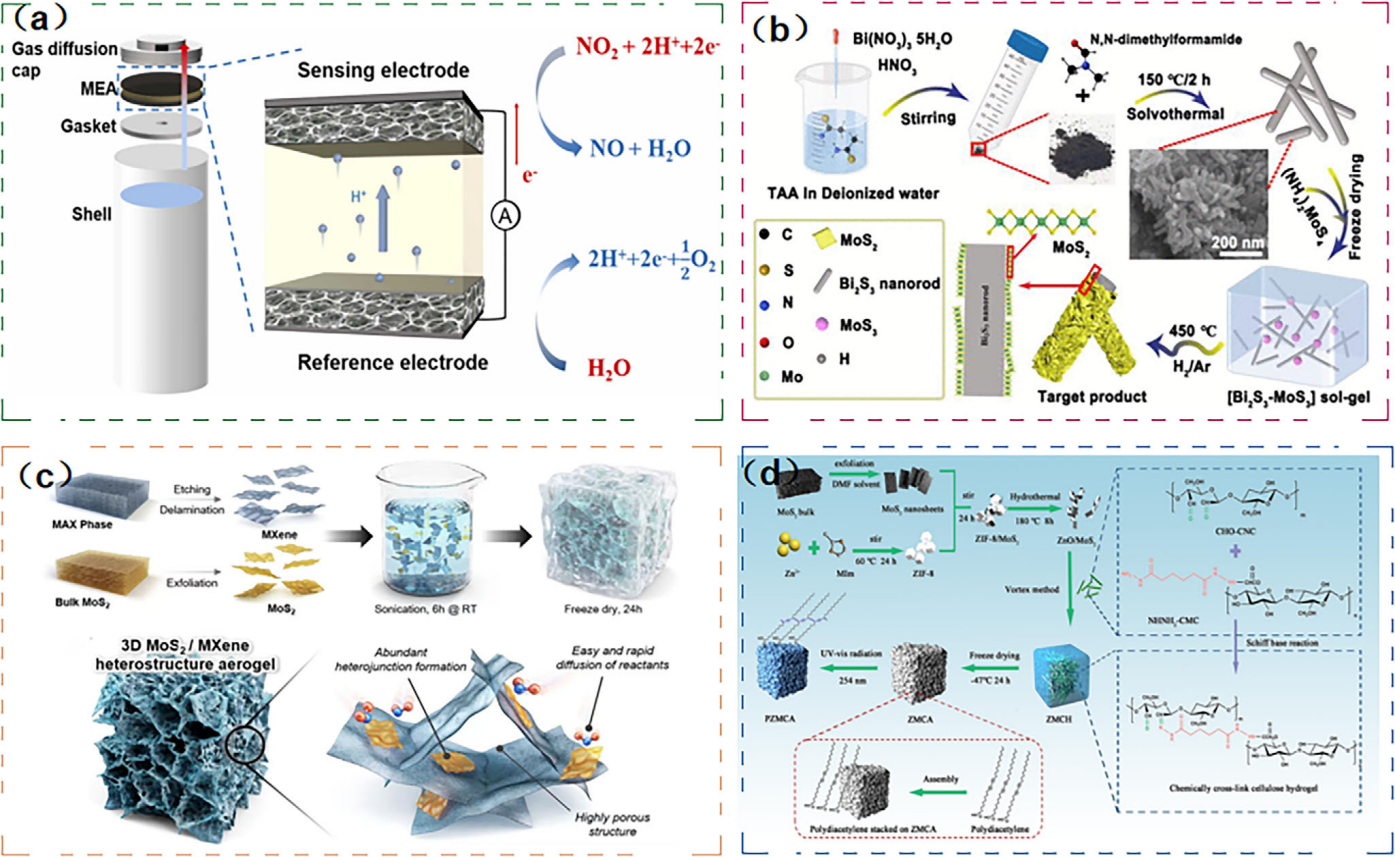
(a) Schematic diagram of the fuel cell‐type gas sensing and NO_2_ sensing mechanisms. Reproduced with permission [90]. Copyright 2025, Elsevier. (b) Schematic diagram of the preparation of the Bi_2_S_3_/MoS_2_ nanocomposite aerogel. Reproduced with permission [91]. Copyright 2023, American Chemical Society. (c) FESEM image of the MoS_2_/MXene nanocomposite aerogel. Reproduced with permission [92]. Copyright 2023, American Chemical Society. (d) Schematic diagram of the PZMCA preparation process. Reproduced with permission [93]. Copyright 2023, Elsevier.

Early research has verified the feasibility of a 3D molybdenum disulfide (MoS_2_) aerogel structure. For instance, Hu et al. [89] directly developed a 3D MoS_2_ composite aerogel for NO_2_ sensing through the freeze‐drying and subsequent thermal annealing of an ammonium thiomolybdate solution. Its optimum operating temperature is 200°C. However, compared to two‐dimensional films prone to stacking, its 3D porous structure ensures continuous accessibility of active sites and rapid gas diffusion. This constitutes the structural basis for maintaining high sensitivity and rapid response‐recovery characteristics. To further enhance performance and expand applications, researchers have developed MoS_2_ aerogels into multifunctional carriers. For instance, Huang et al. [90] used MoS_2_ aerogel as the core carrier of a fuel cell‐type gas sensing material, loading it with a highly active CuS catalyst to create a CuS/MoS_2_ aerogel. The response current to 50ppm NO_2_ at room temperature is as high as 2867.8 nA, and the material exhibits excellent repeatability and moisture resistance. This superior performance stems from the aerogel carrier's large specific surface area (15.1 m^2^/g), which provides ample space for catalytic reactions. Meanwhile, the 3D conductive network ensures the efficient collection and transmission of electrons (Figure 5a). This work first proposed the concept of a multifunctional carrier that integrates catalytic performance with structural support performance, providing a breakthrough strategy for designing novel sensing materials. His work introduces for the first time the concept of multifunctional carriers that integrate catalytic performance with structural support capabilities, providing a breakthrough strategy for designing novel sensing materials.

After developing MoS_2_ aerogel into a multifunctional carrier to enhance its performance, constructing heterojunctions has become a key strategy for optimizing its electronic structure and surface chemical activity further, as well as strengthening its gas sensing performance. This process allows the electronic structure and surface chemical activity of the MoS_2_ aerogel to be optimized systematically. Liu et al. [91] produced a Bi_2_S_3_/MoS_2_ nanocomposite aerogel through freeze‐drying, with large‐sized MoS_2_ nanosheets arranged in parallel on the surface of Bi_2_S_3_ nanorods (Figure 5b). Notably, the formation process of the aerogel itself generates sulfur vacancies on the MoS_2_ surface, and the aerogel's porous and loose structure provides sufficient exposure space and gas contact channels for these defect sites. Consequently, the combined effect of the heterojunction interface charge transfer and the aerogel's structural properties significantly enhances this composite material's response to NO_2_. Kim et al. [92] constructed a 3D MoS_2_/MXene van der Waals heterojunction aerogel via physical mixing and freeze‐drying (Figure 5c). The key to this system is that the MoS_2_ layer effectively inhibits the formation of nitrite on the MXene surface, favoring physical adsorption as a more favorable adsorption mode, while maintaining sufficient charge transfer levels, and ensuring excellent gas sensing response performance. In addition, Fu et al. [93] developed a MoS_2_/ZnO/ZIF‐8 composite aerogel (PZMCA) that integrates detection and adsorption. This work involves constructing heterojunctions using a low‐temperature hydrothermal method, which consumes less energy than the calcination methods commonly used (Figure 5d). Subsequently, polydiacetylene self‐assembled on the aerogel surface to form a flower‐like structure. This microstructure greatly promotes the rapid identification and capture of VOCs.

#### 2.2.2 Cadmium Sulfide Aerogel

As a typical II‐VI group n‐type semiconductor, cadmium sulfide (CdS) possesses a direct bandgap of approximately 2.4 eV, thus garnering significant attention in the field of sensing and detection [94, 95, 96]. However, unlike materials such as SiO_2_, which tend to form aerogels during hydrolysis, CdS nanoparticles exhibit slow kinetics and poor structural stability during sol‐gel conversion. This has led to relatively slow progress in preparing CdS aerogels, which has significantly limited their in‐depth exploration in gas sensing [97].

**FIGURE 6:**
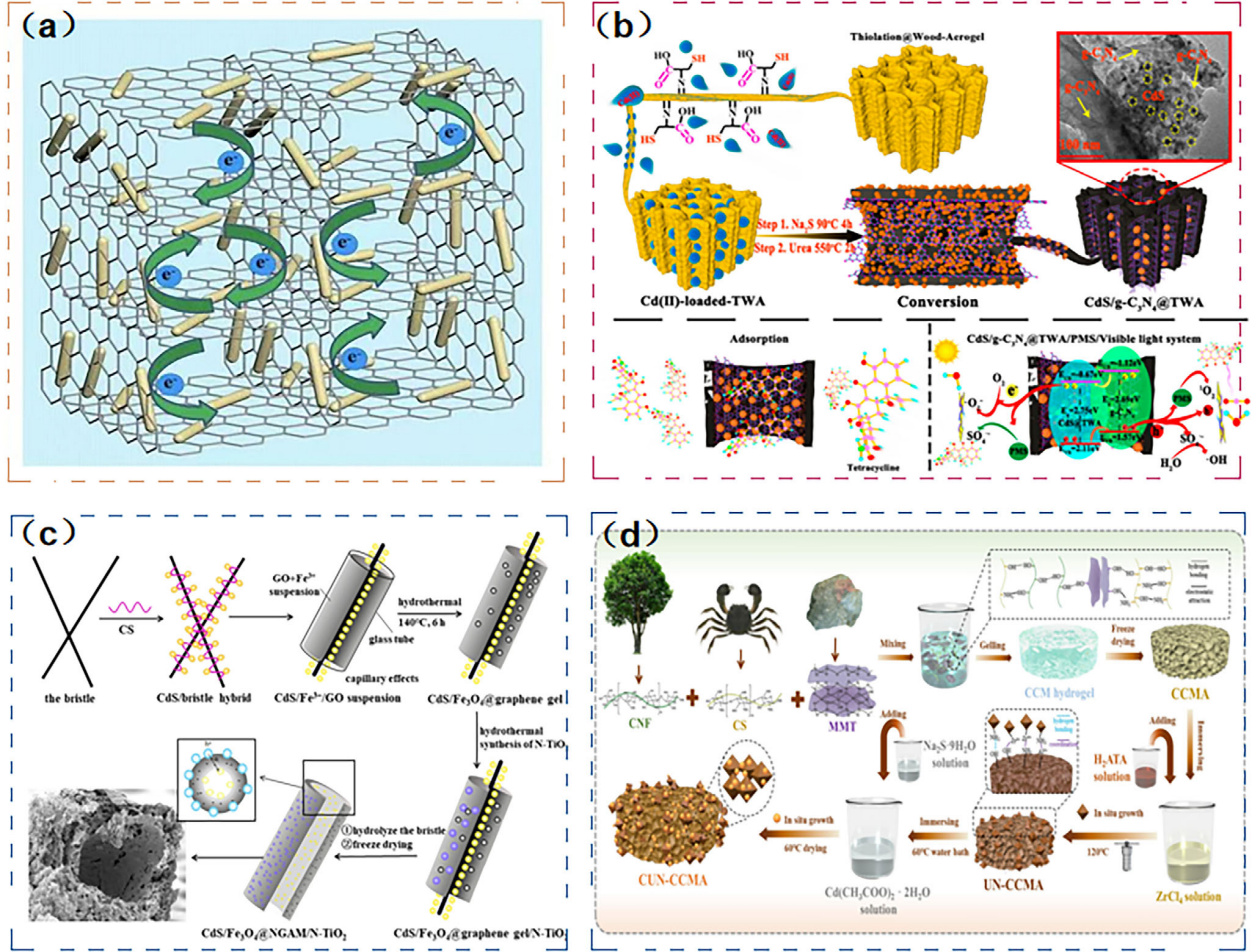
(a) Schematic structure of the 3D RGO/CdS aerogel network. Reproduced with permission [98]. Copyright 2020, Springer nature. (b) in situ construction of the CdS/g‐C_3_N_4_ heterojunction. Reproduced with permission [101]. Copyright 2024, American Chemical Society. (c) Synthesis route of the CdS/Fe_3_O_4_@NGAM/N‐TiO_2_. Reproduced with permission [102]. Copyright 2024, Springer nature. (d) schematic diagram of the CUN‐CCMA preparation process Reproduced with permission [103]. Copyright 2025, Elsevier.

Despite the difficulties involved in preparing pure‐phase CdS aerogels directly, researchers have successfully developed a range of high‐performance CdS‐based aerogel materials using composite strategies. For instance, Cao et al. [98] created a 3D porous RGO/CdS nanowire aerogel using a two‐step process involving the embedding of one‐dimensional CdS nanowires as structural spacers within a graphene network (Figure 6a). This design effectively inhibits the stacking of graphene sheets to form a stable, 3D, interconnected pore structure and significantly increases the material's specific surface area, providing efficient transport channels and abundant surface adsorption sites for gas molecules. This demonstrates its potential application in gas sensing. Regarding gelation, Gacoin et al. [99] highlighted that the key to achieving controlled synthesis of CdS aerogels hinges on regulating the chemical state of the surface to encourage directional condensation. This process necessitates precise control of parameters such as precursor concentration and pH value. Addressing the common challenge of colloidal stability in chalcogenides, Lihitkar et al. [100] innovatively synthesized thiolate‐capped CdS nanoparticles using a microemulsion method, introducing them into silica sol. This method utilizes the strong interaction between the thiolate anions and the silica network to enhance the structural stability of the CdS component effectively, successfully preparing CdS‐doped silica composite aerogels, providing a new approach for the controllable preparation of such materials.

It is important to note that the inherent chemical instability of CdS material poses a significant challenge to their practical application. Constructing heterojunctions has therefore become a key strategy to enhance their stability and optimize its functional properties simultaneously. Zhang [101] and Wang [102] constructed CdS/g‐C_3_N_4_ and CdS/N‐TiO_2_ heterojunctions, respectively, utilizing porous aerogels as functional carriers. This effectively promoted interface separation and the rapid transport of carriers, while also significantly improving the material's chemical stability under harsh conditions (Figure 6b,c). Mi et al. [103] formed a composite aerogel (CUN‐CCMA) loaded with CdS‐based heterojunctions through solvothermal synthesis and water bath heating (Figure 6d). The aerogel carrier is lightweight (density of 0.056 g/cm^3^), has a high specific surface area (52.399 m^2^/g) and a wide pore size (1–379 nm, mainly micropores), and provides a 3D porous structure that enhances mechanical stability and regulates the uniform dispersion of heterojunctions. These achievements demonstrate the potential for developing a new generation of CdS‐based gas sensing materials with high sensitivity, excellent selectivity, and long‐term stability through the synergistic design of heterojunction engineering and macroscopic 3D structure.

### 2.3 Carbon‐Based Aerogel

#### 2.3.1 Graphene Aerogel

As a typical single‐atom‐layer, two‐dimensional material, graphene has garnered significant attention since its first successful preparation [104, 105]. It exhibits excellent electron transport properties, an ultra‐high specific surface area, superior carrier mobility, good mechanical strength, and chemical stability. These characteristics collectively make graphene an ideal material for constructing high‐performance gas sensors [106, 107]. During the sensing process, gas molecules can adsorb onto the graphene surface as electron donors or acceptors. This effectively modulates the carrier concentration and induces significant changes in resistance, providing a basis for the sensitive detection of various gases [108]. However, graphene aerogels still face severe challenges in practical gas sensing applications. The inability to differentiate between various gases results in poor selectivity of the sensing materials. The weak physical adsorption between gas molecules and the graphene surface often leads to a slow desorption process and a delayed response recovery rate. Furthermore, it exhibits insufficient long‐term stability under actual environmental conditions [109, 110].

**FIGURE 7:**
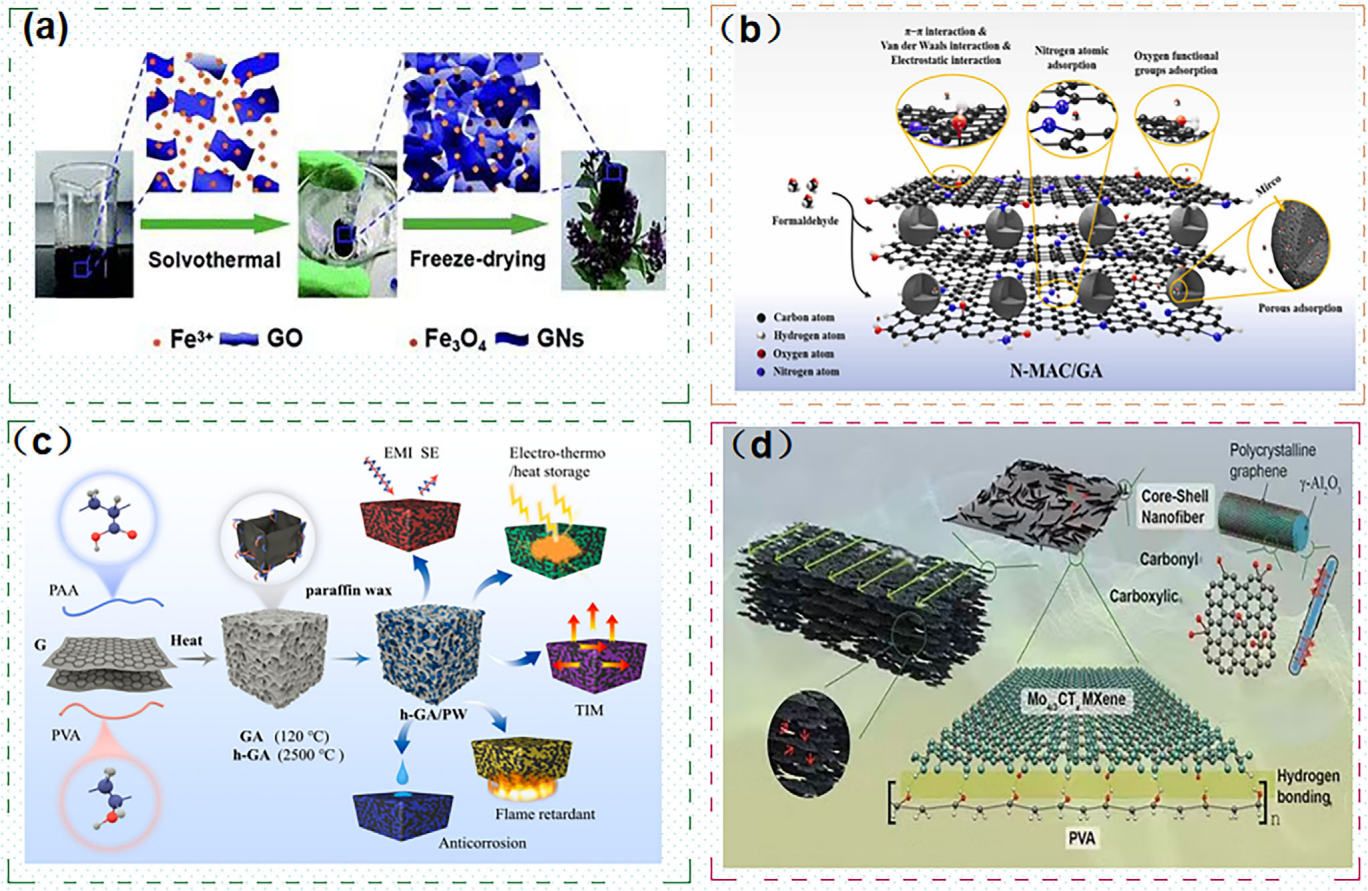
(a) Manufacturing process of 3D Fe/GAs nanocomposite material. Reproduced with permission [111]. Copyright 2015, RSC Publishing. (b) Schematic diagram showing the possible interaction between N‐MAC/GA and formaldehyde molecules, Reproduced with permission [113]. Copyright 2025, Elsevier. (c) Schematic diagram showing the preparation process of graphene aerogel composite material. Reproduced with permission [114]. Copyright 2025, Elsevier. (d) Schematic diagram showing the preparation process of Mo_4_/_3_CTX MXene aerogel. Reproduced with permission [115]. Copyright 2025, American Chemical Society.

To overcome the above bottlenecks, researchers have developed various functionalization and composite strategies. These aims to optimize structural stability and gas sensing performance synergistically by constructing a 3D composite aerogel system. The combination of graphene and metal oxide nanoparticles is particularly effective in enhancing the material's conductivity, specific surface area, and number of active sites. For example, Liu et al. [111] synthesized a composite material comprising a 3D graphene aerogel loaded with Fe_3_O_4_ nanoparticles. This material exhibits a macroscopically interconnected microporous network framework, within which the Fe_3_O_4_ nanoparticles are uniformly dispersed. The ultra‐high specific surface area of the graphene aerogel provides many active sites and facilitates the rapid diffusion of target gases, resulting in the material's excellent sensing performance. The response and recovery times are 150 and 113 s, respectively, and the effective detection concentration of the target gas is as low as 0.1 ppm (Figure 7a).

However, this type of composite material generally still faces issues such as slow recovery rates in practical applications, and further optimization is needed. To address this, researchers have promoted the optimization of aerogel sensing performance through enhanced preparation methods and structural modifications. For instance, Bibi et al. [112] systematically investigated the room‐temperature sensing performance of PANI/GO aerogel (PGA) toward H_2_S gas, achieving a response time of merely 21 s. This sensing material not only demonstrated high sensitivity but also performed consistently well in repetitive tests, with the dynamic adsorption/desorption curves of three consecutive tests almost perfectly overlapping, thereby demonstrating stable response values and the material's excellent reversibility. Xu et al. [113] prepared a nitrogen‐doped, modified activated carbon/graphene composite aerogel (N‐MAC/G) using the hydrothermal method. Graphene forms a 3D framework through π‐π stacking, enhancing the chemical adsorption of formaldehyde (Figure 7b). This aerogel has a specific surface area of 621.2 m^2^/g and a hierarchical porous structure of 2–50 nm. Its response sensitivity to 10–1000 ppm of formaldehyde is 4.2 times higher than that of pure graphene aerogel, with response and recovery times reduced to 8 and 25 s, respectively. The abundant polar functional groups on the surface also facilitate subsequent functionalization modifications.

In addition to optimizing the gas sensing performance of aerogels for sensing applications, researchers have focused on expanding the compatibility of the preparation process and the structural and functional diversity of graphene aerogels. They have also explored their application potential in multiple scenarios through innovative synthesis methods and composite system design. Meng et al. [114] developed an environmentally friendly method of preparing graphene aerogels using electrochemically exfoliated graphene as the raw material. Through the condensation‐dehydration reaction of hydrophilic polymers (such as polyvinyl alcohol), they constructed a 3D, lightweight aerogel (density 0.12 g/cm^3^) at room temperature. The strong interfacial coupling between graphene sheets and polymer/carbonization residues, formed by hydrogen bonding and van der Waals forces, endows the material with high performance (Figure 7c). Shamshirgar et al. [115] prepared a graphene‐enhanced alumina nanofiber (GAIN)/polyvinyl alcohol (PVA) hybrid aerogel using a bidirectional freeze‐drying method. Subsequently, GAIN and PVA solutions were mixed and, after bidirectional freezing and freeze‐drying, a hybrid aerogel formed (Figure 7d). This aerogel exhibits an anisotropic layered structure with a porosity of 93% (pore size 10–50 µm) in the uncompressed sample. After compression at 10 MPa, the porosity decreased to 20%–23%, while the compression modulus increased to 15 MPa (eight times that of the uncompressed sample). The introduction of graphene also provides conductive pathways for the aerogel.

#### 2.3.2 Carbon Nanotube Aerogel

Carbon nanotubes (CNTs) are considered one of the most promising materials for miniaturized sensors thanks to their unique electronic surface properties, high surface area, and sensitivity to target molecules [116, 117, 118]. Assembling CNTs into a 3D aerogel structure effectively overcomes the tendency of nanomaterials to agglomerate, while leveraging their advantages, such as a large specific surface area, a tunable pore structure, and high conductivity. This provides an excellent platform for constructing high‐performance chemical sensing materials [119, 120, 121].

**FIGURE 8:**
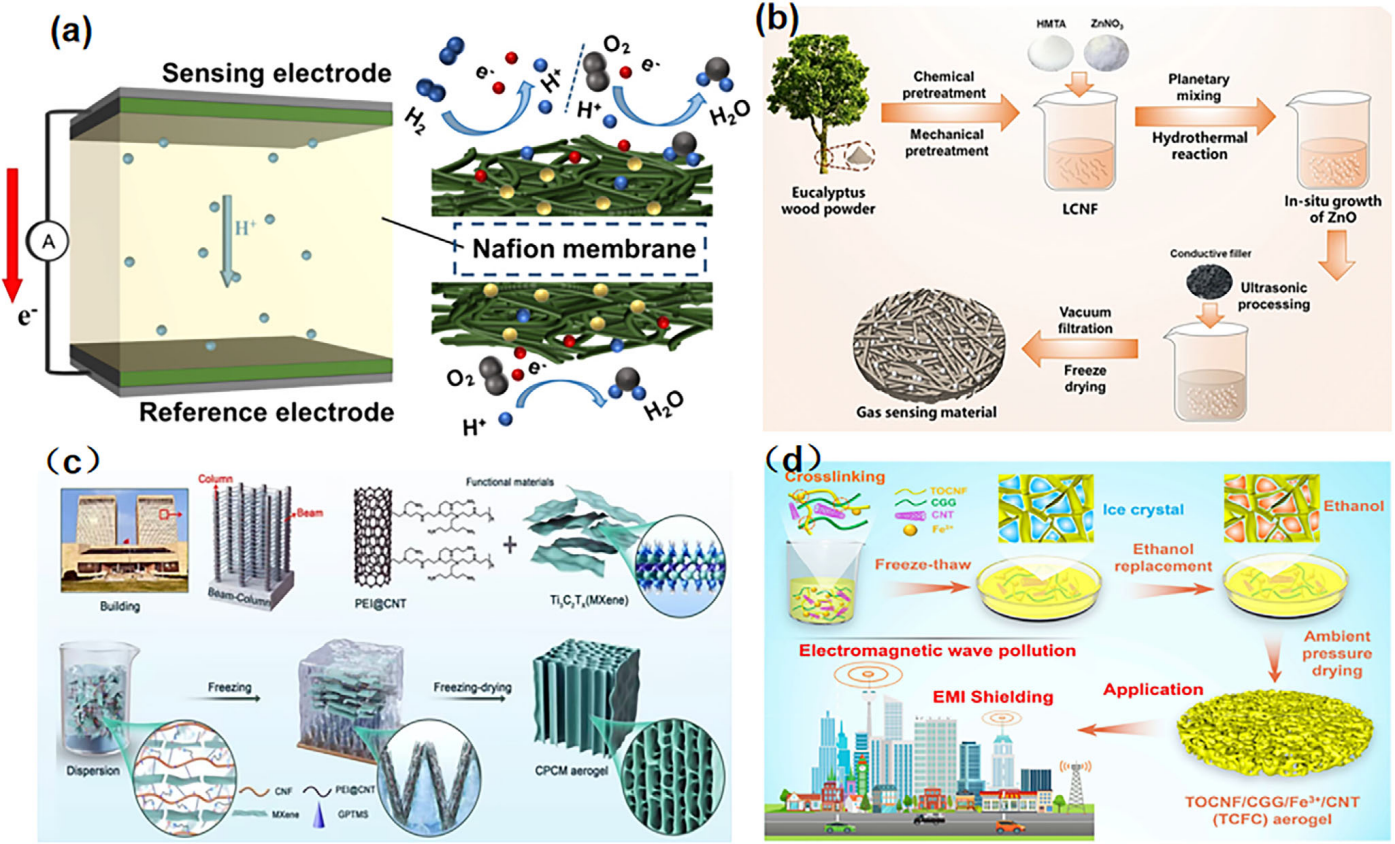
(a) Schematic diagram of the sensing mechanism of a fuel cell‐type hydrogen sensor. Reproduced with permission [123]. Copyright 2025, Springer nature. (b) Schematic diagram of the synthesis process of LCNF/ZnO composite aerogel. Reproduced with permission [124]. Copyright 2024, American Chemical Society. (c) Preparation flowchart of CPCM aerogel. Reproduced with permission [125]. Copyright 2025, Elsevier. (d) Schematic diagram of the preparation and application of TCFC aerogel. Reproduced with permission [126]. Copyright 2025, Elsevier.

In resistive gas sensing materials, the catalyst and its carrier material jointly determine the upper limit of sensing performance. Traditional electrode materials often rely on combinations of noble metal catalysts and carbon carriers. However, these materials exhibit limited catalytic activity toward certain gases (such as SO_2_) and are susceptible to decreased proton conductivity. Research has shown that combining CNTs with copper (Cu), phytic acid, and other materials to form an aerogel structure can offer significant advantages in addressing these challenges. For example, Huang et al. [122] developed a novel SO_2_ sensing aerogel. This aerogel uses the 3D CNT structure to disperse catalytic sites and enhance gas diffusion and electron transport. Furthermore, doping CNT aerogels with heteroatoms can precisely tune their surface electronic structure, effectively enhancing their intrinsic sensing performance. Huang et al. [123] prepared a phosphorus‐doped CNT aerogel (Figure 8a) for hydrogen detection by mixing CNTs with phytic acid. The aerogel exhibited a high response value of 921.9 µA at 15 000 ppm of hydrogen, with a sensitivity of −0.063 µA/ppm. This sensing material maintains stable performance in different humid environments.

To propel CNT aerogels from their outstanding material properties toward substantive breakthroughs in practical device applications, the precise, controllable formation and long‐term stable maintenance of their macrostructure have become core challenges requiring urgent resolution. Due to problems such as easy collapse and insufficient structural strength during aerogel preparation, research has shifted toward improving formability, structural integrity, and durability simultaneously through sophisticated microstructure design. This involves building chemical cross‐linking networks, adopting new drying processes, and creating bionic multi‐stage structures. For example, Li et al. [124] designed a biodegradable lignocellulose nanofiber (LCNF)/ZnO/CNT composite aerogel (Figure 8b). In this system, the introduction of a small amount of CNTs created efficient electronic conduction pathways within the aerogel, significantly improving its response to ammonia (NH_3_) at room temperature. Furthermore, the aerogel demonstrated exceptional mechanical elasticity, recovering 87.5% of its thickness after withstanding a load 1000 times its own weight. Xu et al. [125] prepared CNF/PEI@CNT/MXene (CPCM) through directional freeze‐drying, resulting in an aerogel with a unique beam‐column structure (Figure 8c). Performance tests showed that the CPCM aerogel exhibited a stress of 25.65 kPa at 50% compressive strain and demonstrated excellent shape recovery and structural stability in multiple cyclic compression‐recovery tests, with minimal plastic deformation. Wei et al. [126] prepared TOCNF/CGG/Fe^3+^/CNT (TCFC) aerogels using a dual‐crosslinking network‐assisted ambient pressure drying strategy. In the dual‐crosslinking network, CNTs not only obtained stable conductive pathways but also significantly enhanced the mechanical strength of the network. This reinforced structure effectively resisted capillary forces during ambient pressure drying, preventing the collapse of the porous skeleton, thus maintaining a complete aerogel morphology even at an extremely low density (0.1071 g/cm^3^) (Figure 8d). The aerogel exhibited high porosity (77.44%) and a large specific surface area (70.15 m^2^/g).

### 2.4 Other Composite Aerogel

#### 2.4.1 MOFs‐Based Aerogel

**FIGURE 9:**
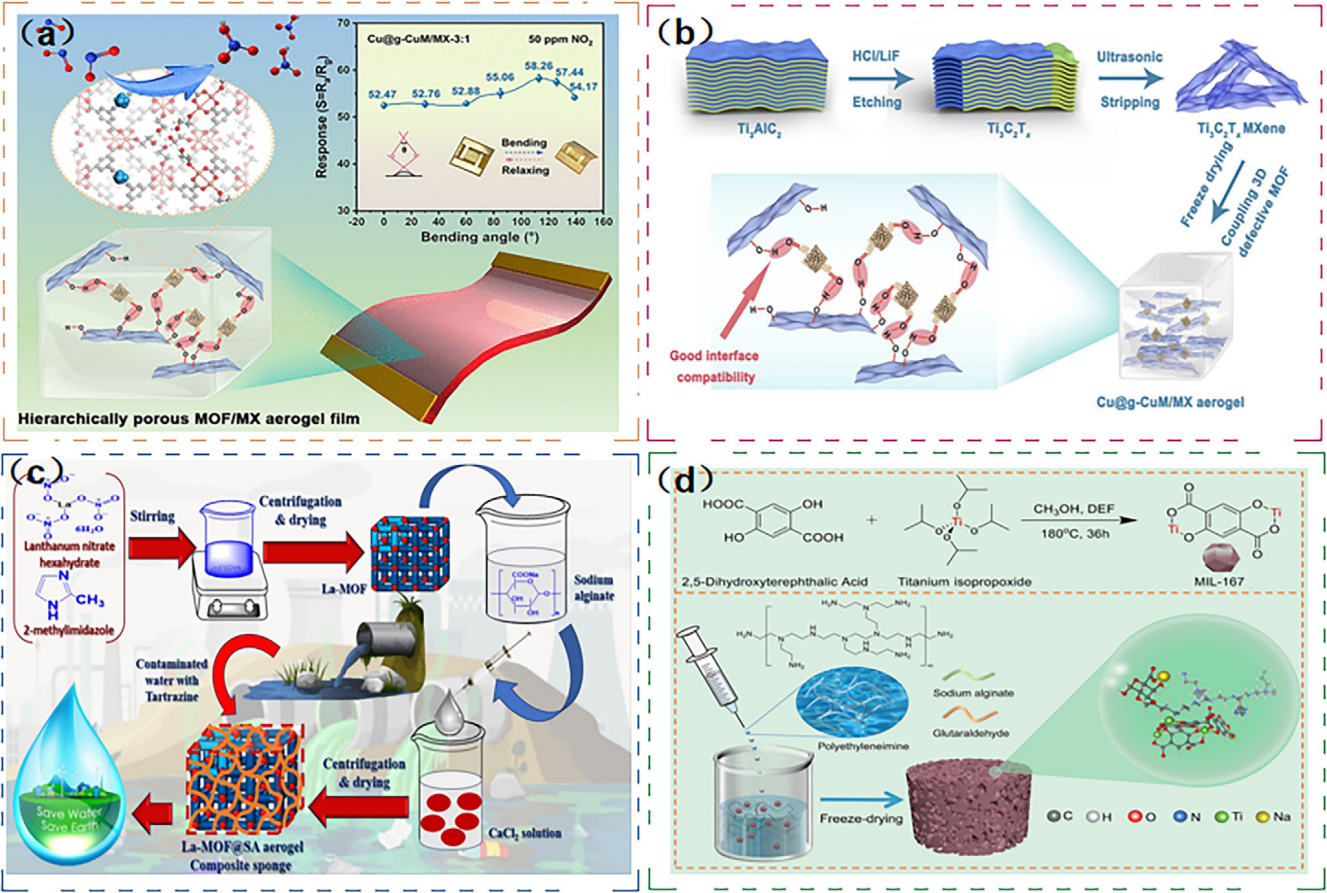
(a) Schematic diagram of the preparation process of MC/CS@MXene aerogel (MC = methyl cellulose, CS = chitosan). Reproduced with permission [133]. Copyright 2024, American Chemical Society. (b) Enlarged view of the preparation process of Cu@g‐CuM/MX aerogel showing the hydrogen bonding interaction between MXene and Cu@g‐CuM. Reproduced with permission (133]. Copyright 2024, American Chemical Society. (c) Schematic diagram of the preparation of La‐MOFs@CA aerogel composite sponge. Reproduced with permission [134]. Copyright 2024, Elsevier. (d) Synthesis schematic diagram of MIL‐167/SA‐PEI. Reproduced with permission [139]. Copyright 2025, Elsevier.

Metal‐organic frameworks (MOFs) are crystalline, porous materials formed by the self‐assembly of metal ions/clusters and organic ligands via coordination bonds. MOFs possess an ultra‐high specific surface area, a tunable pore structure, and an abundance of metal active centers. They have shown great potential for use in catalysis, sensing, and biomedicine [127, 128, 129]. However, MOFs suffer from poor intrinsic conductivity and insufficient availability of active sites. They are also susceptible to agglomeration in powder form. These factors severely restrict their direct application in gas sensing and make it difficult to meet the requirements of high‐performance sensing materials in terms of charge transfer efficiency and structural stability [130, 131, 132]. Integrating MOFs with functional materials is a core strategy for overcoming performance limitations and achieving functional integration. For instance, Liu et al. [133] proposed a thermally induced, solvent‐assisted oxygen anion etching strategy. By precisely controlling the structure and framework defects of the MOFs, the metal active sites were fully exposed, and the MOFs self‐assembled to form a conductive copper‐based metal‐organic framework (CuMOF)‐based composite aerogel (Figure 9a,b). This sensing material exhibits no significant performance degradation after 500 bending cycles, combining excellent gas sensing performance with mechanical durability. Its core advantage stems from the specific adsorption effect of etching defects.

To improve the mechanical stability of materials, they can be compounded with biopolymers. For example, Mohamed et al. [134] combined La‐MOFs with sodium alginate (CA) (Figure 9c). The 3D, cross‐linked CA network provides stable support for the MOF particles, significantly inhibiting agglomeration and structural collapse. Meanwhile, the MOFs are highly dispersed throughout the network, giving the composite material a specific surface area of up to 1244.68 m^2^/g and uniformly distributed active sites. This synergistic effect enhances the overall structural stability and potentially improves the catalytic oxidation efficiency of sensing gases through electronic regulation between ligands and metal centers.

In addition to effectively improving the mechanical properties, researchers have also increased the number and accessibility of active sites by introducing ligand defects and constructing porous MOFs, thereby optimizing the performance of MOF‐based materials further [135, 136, 137]. For instance, Mai et al. [138] used a surfactant template‐assisted solvothermal method to grow many mesoporous crystals within the material in situ, creating a 3D MOF‐polymer composite aerogel. This material has a specific surface area of up to 900 m^2^/g, can withstand a stress of approximately 40 kPa under a compression strain of 70%, and is therefore suitable for real‐time ammonia monitoring in wearable devices. Its amino functional groups can also form specific hydrogen bonding interactions with ammonia. Yuan et al. [139] prepared an MOF‐based composite aerogel (MIL‐167/SA‐PEI) using the sol‐gel method (Figure 9d). This material has a spongy, loose, and porous structure with a highly interconnected 3D network that can dissipate external stress effectively. It also has excellent reversible compressibility and can withstand a stress of approximately 975 kPa under a compression strain of 85%.

#### 2.4.2 Mxene Aerogel

MXene is a new class of two‐dimensional transition metal carbides, nitrides, and carbonitrides that exhibit notable properties, including high conductivity, strong hydrophilicity, and tunable surface chemistry. In recent years, its extensive application potential has been demonstrated in various fields, including lithium‐ion batteries, energy storage systems, electronic devices, biosensors, and transparent conductors. The abundance of terminal groups (such as ‐O, ‐OH, and ‐F) and the high conductivity of MXene provide it with numerous active sites for gas adsorption, making it a highly promising gas sensing material.

**FIGURE 10:**
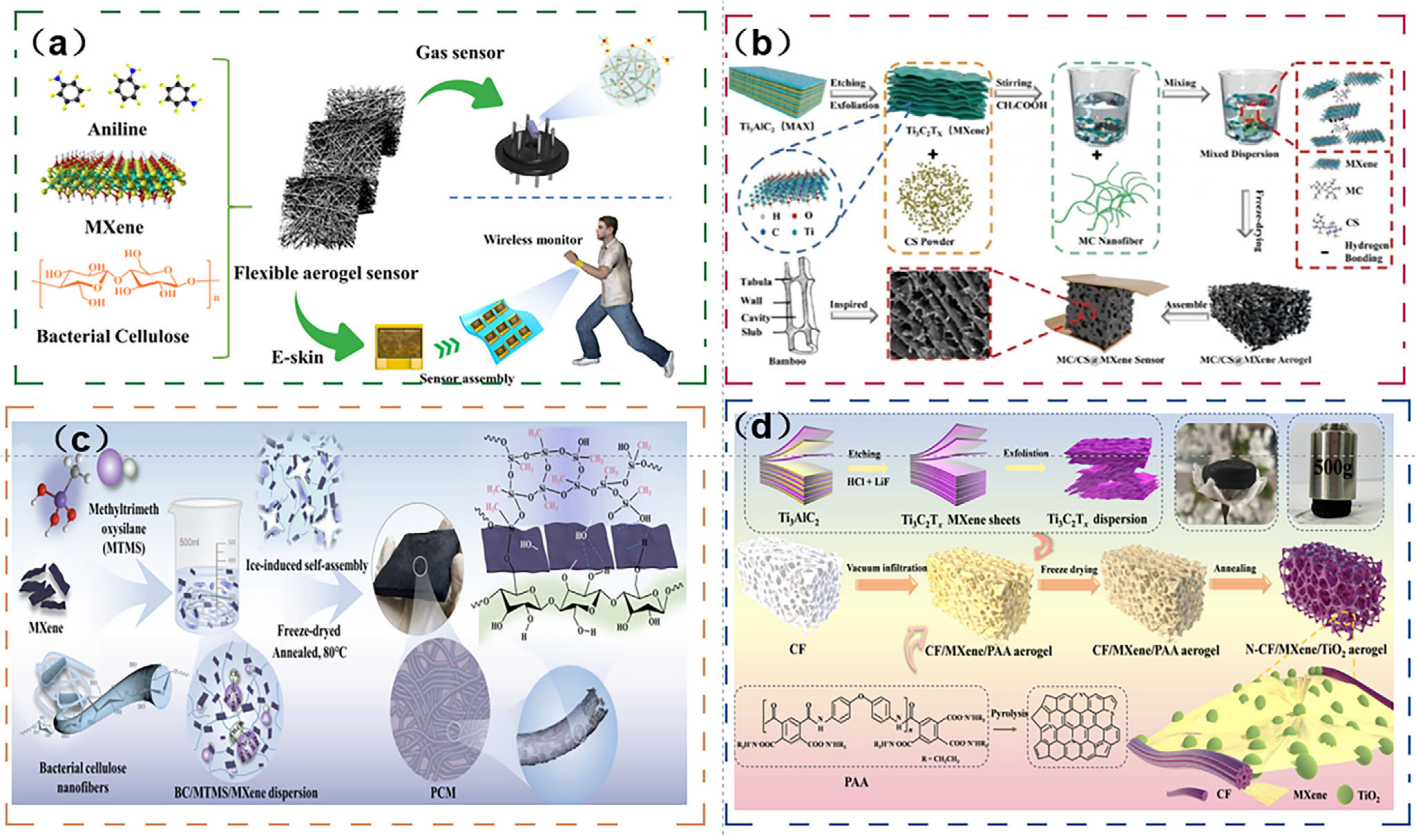
(a) A schematic diagram showing the components of the MXene/PANI/BC aerogel and its applications in electronic skin and gas sensors. Reproduced with permission [140]. Copyright 2021, American Chemical Society. (b) Schematic of the MC/CS@MXene aerogel preparation process (MC = methylcellulose; CS = chitosan). Reproduced with permission [142]. Copyright 2025, Springer Nature. (c) A schematic diagram showing the preparation process. Reproduced with permission [144]. Copyright 2023, Elsevier (d) A schematic diagram showing the preparation process of the 3D N‐CMT nano‐gel. Reproduced with permission [146]. Copyright 2025, Tsinghua University Press.

To make the most of MXene's excellent properties, researchers have created a series of high‐performance MXene composite aerogels by combining it with other functional materials. Zhi et al. [140] successfully produced MXene/PANI/BC composite aerogels using a self‐assembly approach (Figure 10a). The 3D porous structure of this material and its abundant terminal groups give it a significant gas sensing response to NH_3_. The hydrogen bonding network formed between the MXene, PANI, and BC components ensures the stability of the overall structure and the reliability of the signal channel. Nie et al. [141] designed an MXene/Au/GO/4‐ATP composite aerogel to enable the rapid detection of aldehydes in construction machinery exhaust fumes. This aerogel uses the conductivity of MXene, the adsorption properties of graphene oxide (GO), and the plasmon resonance effect of gold nanoparticles to enable high‐sensitivity and high‐selectivity detection of aldehydes, even in complex exhaust environments. Zhao et al. [142] prepared MC/CS@MXene composite aerogels by physically blending the materials and freeze‐drying them (Figure 10b). During formation, the material constructs a multi‐level structure resembling the internal cavity of bamboo. In this structure, hydrogen‐bonded MC/MXene acts as ′bricks″ to provide support, while electrostatic adsorption between CS and MXene acts as ′mortar″ to fill the gaps. This significantly enhances the stability of the overall structure. This ′brick‐mortar″ assembly mode tightly connects the porous layers, forming a stable 3D conductive network. This excellent mechanical sensing performance provides a solid basis for developing wearable gas sensors based on cellulose aerogels that can withstand deformation. Wu et al. [143] used in situ growth and unidirectional freeze‐drying techniques to fabricate MXene/BC@CoFe_2_O_4_ aerogels. These aerogels have a unidirectional porous structure, with MXene sheets acting as the inner walls of directional pores to enhance their support strength. The compressive stress of this aerogel increased 3.6‐fold to 110 kPa, demonstrating its robust mechanical properties and great potential for gas sensing applications.

The above research successfully overcame the issue of insufficient mechanical stability in materials by providing a robust and durable substrate for gas sensors. However, structural reinforcement alone is insufficient to achieve high sensitivity and selectivity in gas detection. Precise design and functionalization regulation at the molecular and interface levels are also required. For example, Liu et al. [133] used a thermally induced, solvent‐assisted oxygen anion etching method to open pores selectively in CuM. The hydrogen bonding interaction between the carbonyl oxygen atoms and the functional hydroxyl groups on the MXene surface then self‐assembled to form a conductive CuM/MXene aerogel. This aerogel exhibited flexible sensing performance for NO_2_. This sensor exhibited excellent sensitivity (a response value of S = 52.47 for 50 ppm NO_2_), good selectivity, and fast response and recovery times (0.9 and 4.5 s, respectively) at room temperature. Zhao et al. [144] prepared a novel poly (methyl sesquisiloxane) (PMSQ)/cellulose/MXene composite aerogel (PCM) (as shown in Figure 10c). This lightweight material has a density of 16.2 mg/cm3 and exhibits excellent mechanical toughness, rapidly recovering its original shape after withstanding a pressure 9000 times its own weight. Xu et al. [145] designed and produced an MXene/PI/Ag nanowire composite aerogel via a bidirectional freezing and annealing process. The directional growth of the precursor under dual temperature gradients resulted in a layered microstructure within the composite aerogel. The close interconnection between the MXene nanosheets and the polyimide (PI) molecular chains enables stress to be transmitted and dispersed uniformly within the composite aerogel, imparting excellent mechanical strength and elastic recovery properties to the material. At the same time, the MXene nanosheets exhibit adjustable interlayer spacing under stress and undergo dynamic changes in their interlayer structure, which further buffers the impact of external forces and enhances the mechanical adaptability and structural stability of the composite aerogel. Li et al. [146] proposed a micro‐macro synergistic strategy to construct a porous MXene‐based aerogel using carbon fibers as the skeleton. By utilizing the easy oxidation characteristics of MXene in combination with polyamide acid, MXene interlayer stacking was effectively suppressed to form a 3D loofah‐like structure. Following thermal treatment, TiO_2_ and carbon layers were generated within the MXene nanosheets and fibers in situ, creating an abundance of heterogeneous interfaces and significantly optimizing the material properties (Figure 10d).

In summary, different types of aerogels exhibit distinct gas‐sensing properties due to differences in material composition, pore structure characteristics, and interfacial configurations. Among these, high specific surface area and interconnected pore channels facilitate gas adsorption and diffusion, while heterojunctions and defect structures can regulate carrier transport and interfacial reaction processes. However, how these structural advantages are ultimately converted into sensing signals still depends on the interaction mechanisms between gas molecules and the material. Therefore, building on the introduction of various aerogel systems and their structural design strategies, the next section will further discuss the fundamental mechanisms of aerogel gas sensing, focusing on how processes such as gas adsorption, electron transfer, and surface reactions influence sensing performance.

## 3 Sensing Mechanism

### 3.1 High‐Temperature Sensing Mechanism

The core principle of resistive aerogels for gas sensing is based on the interaction between the target gases and the gas‐sensitive materials. This interaction induces changes in the materials ′ conductivity [147]. The gas sensing process can be divided into three stages: gas diffusion, gas‐solid interface reaction, and charge carrier migration.

During the gas diffusion stage, oxygen molecules and target gas molecules must be transported to the surface of the gas‐sensing material via molecular diffusion, surface diffusion, or Knudsen diffusion. Since aerogel materials usually have pore sizes in the range of 1–100 nm, gas molecules mainly penetrate the interior of the aerogel through Knudsen diffusion [148]. When these molecules reach the surface of the aerogel gas‐sensing layer in the aerogel, the gas‐solid interface reaction stage begins. First, thermal excitation causes some electrons in the semiconductor material to transition from the valence band to the conduction band. Subsequently, oxygen in the air undergoes physical or chemical adsorption on the material surface, capturing electrons in the conduction band and forming oxygen anions [149]. This charge transfer process alters the electron concentration on the semiconductor surface. This leads to the formation of a built‐in electric field perpendicular to the grain surface. This causes the energy band to bend upward and simultaneously forms a space charge layer and potential barrier in the surface region. If the semiconductor component in the aerogel material is n‐type, the space charge region manifests as an electron depletion layer (EDL), and if it is p‐type, the space charge region manifests as a hole accumulation layer (HAL) [150, 151]. The potential barrier height (Vs) and the width of the space charge region (X) can be approximately derived through Poisson's equation [152]:

(1) $$Vs=\frac{q^{*}N_{S}^{2}}{2^{*}\varepsilon_{S}^{*}\varepsilon_{0}^{*}N_{D}}$$

(2) $$x=\sqrt{\frac{2^{*}\varepsilon_{S}^{*}Vs}{q^{*}N_{D}}}$$

In the formula, q represents the charge of an electron, ε_s_ denotes the dielectric constant of the semiconductor, ε_0_ signifies the dielectric constant of vacuum, N_s_ stands for the net ion density on the semiconductor surface (the density of adsorbed oxygen anions), and N_D_ indicates the electron donor density (the density of oxygen defects in the semiconductor). Once oxygen has attained dynamic adsorption equilibrium, the semiconductor material begins to interact with the target gas.

In aerogel‐based gas sensors, the type of semiconductor material determines its response mechanism. For p‐type semiconductors, when the target gas is a reducing gas such as hydrogen, it reacts with the oxygen species adsorbed on the surface of the material, consuming holes or introducing electrons. This ultimately leads to the weakening of the hole accumulation layer and a decrease in the conductivity of the material. If the target gas is an oxidizing gas such as NO_2_, it will further capture electrons or generate holes, thereby enhancing the hole accumulation effect and increasing the conductivity of the material [153]. The response pattern of n‐type semiconductors is the opposite, reducing gases can inject electrons into the material, usually increasing conductivity, while oxidizing gases extract electrons from the material, decreasing conductivity [154].

The final stage of gas sensing involves charge carriers migrating within the crystal grains. When an external direct current voltage is applied, charge carriers in semiconductor materials migrate in a specific direction within the crystal grains, generating a current in the circuit. Under constant voltage conditions, the magnitude of the circuit current depends primarily on the resistance of the semiconductor thin film. According to Boltzmann's distribution law, the equivalent resistance (R) of an n‐type aerogel semiconductor satisfies the following relationship [155]:

(3) $$R=R_{0}*\exp\left(\frac{V_{S}}{k*T}\right)$$

**FIGURE 11:**
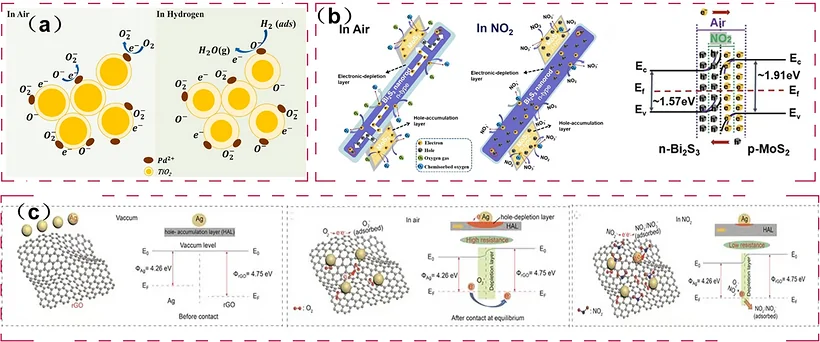
(a) Schematic diagram of the gas sensing mechanism of TiO_2_ composite aerogel. Reproduced with permission [79]. Copyright 2024 Elsevier. (b) Schematic diagram of the possible sensing mechanism and surface processes of Bi_2_S_3_/MoS_2_ heterojunction in air and NO_2_, and Band diagram of n‐type Bi_2_S_3_ and p‐type MoS_2_ in contact with each other in air and NO_2_ gas, NO_2_ sensing mechanism of Ag‐rGO. Reproduced with permission [91]. Copyright 2023 American Chemical Society. (c) Band diagram of Ag and rGO before contact in vacuum, Band diagram of Ag‐rGO composite material after contact in air and Band diagram of Ag‐rGO composite material after contact in NO_2_. Reproduced with permission [167]. Copyright 2020 American Chemical Society.

In the formula, R_0_ represents the initial resistance, V_s_ denotes the barrier height, k stands for the Boltzmann constant, and T signifies the interface temperature. As can be seen from the formula, during oxidation reactions on the surface of n‐type semiconductor aerogels, the potential barriers formed at grain boundaries hinder electron transport, leading to an increase in resistance. When exposed to reducing gases, the barrier height established by pre‐adsorbed oxygen decreases, facilitating smoother electron transport and subsequently reducing resistance [156]. Conversely, p‐type semiconductor aerogels exhibit the opposite behavior: resistance increases in reducing gases and decreases in oxidizing gases. The magnitude of the change in resistance is highly correlated with the concentration of the target gas, enabling the quantitative detection of gas concentration through changes in resistance before and after the reaction [157]. For instance, TiO_2_ composite aerogels prepared by Sui et al. [79] demonstrate excellent hydrogen sensing performance. An increase in oxygen defects leads to an increase in oxygen adsorption sites, effectively promoting hydrogen dissociation. The abundant presence of O^−^ and O_2_^−^ in the material framework reacts with adsorbed hydrogen, releasing many electrons. This reduces the material's resistance and increases the concentration of carriers, significantly enhancing sensitivity (Figure 11a). The optimal operating temperature is 325°C, with a response value of 4.8 to 100 ppm of hydrogen. Advantages include a short response time (<2 s), fast recovery speed (<22 s), high selectivity, and long‐term stability.

### 3.2 Room Temperature Sensing Mechanism

Once gas molecules have reached the material surface through diffusion, the sensing process enters a crucial stage involving surface interaction. At room temperature, the interaction mechanisms between gas molecules and sensitive materials fall into one of four categories: the oxygen adsorption mechanism, the charge transfer mechanism, the equivalent circuit mechanism, and the electron scattering mechanism. The core differences between these mechanisms lie in the nature of the interaction (physical or chemical adsorption) and whether charge is transferred or a chemical reaction occurs. Together, they determine the response direction, amplitude, and dynamic behavior of the sensing material. Below, we will elaborate on these three typical mechanisms separately.

#### 3.2.1 Oxygen Adsorption Mechanism

The oxygen adsorption mechanism is one of the most fundamental and widely applicable mechanisms in the field of gas sensing. Its core logic is closely related to the electron transfer characteristics of material surfaces, and it is widely used in various types of semiconductor sensing materials [158]. When aerogel composite materials are exposed to air, their surfaces have a high specific surface area and abundant active sites and surface defect structures. This provides favorable conditions for the adsorption of oxygen molecules. O_2_ molecules in the air rapidly diffuse to the surface of the sensing material and undergo physical adsorption, which gradually transitions to chemical adsorption. This ultimately forms various stable oxygen species (O_2_^−^, O^−^, and O_2_^−^) on the material surface [61, 159].

This oxygen adsorption mechanism is applicable not only to n‐type aerogel semiconductor materials but also to p‐type aerogel semiconductor materials, where it can explain the sensing response process. The main difference between the two is the direction of electron transfer and how surface carrier concentration changes. In n‐type aerogel semiconductors, there are many free electrons in the conduction band. When exposed to air, oxygen molecules adsorbed on the surface capture the free electrons in the conduction band due to their strong electron affinity, thus completing their ionization process [160, 161]. In contrast, p‐type aerogel semiconductors have many holes in the valence band. The adsorption of oxygen molecules promotes the transfer of holes from the valence band to the surface and facilitates the ionization of the oxygen molecules. The adsorption of surface oxygen in both types of semiconductors will change the carrier concentration of the material, leading to significant changes in material resistance [162].

Specifically, the adsorption and ionization process of oxygen molecules on the surface of sensing materials follows a stepwise reaction pattern, with different reaction steps corresponding to the generation of different oxygen species. Equations (4), (5), (6), (7) provide a detailed description of this complete reaction process [163]:

(4) $$O_{2}\left(\mathrm{gas}\right)\to O_{2}\left(\mathrm{ads}\right)$$

(5) $$O_{2}\left(\mathrm{gas}\right)+e^{-}\to {O_{2}}^{-}\left(\mathrm{ads}\right)$$

(6) $$O_{2}-\left(\mathrm{gas}\right)+2e^{-}\to 2O^{-}\left(\mathrm{ads}\right)$$

(7) $${O_{2}}^{-}\left(\mathrm{gas}\right)+4e^{-}\to 2{O_{2}}^{-}\left(\mathrm{ads}\right)$$

The adsorption of oxygen and the subsequent ionization process form the basis for the creation of stable surface states and initial resistance in many semiconductor aerogel materials in an air environment. Stable surface oxygen species form an EDL or HAL layer on the material surface, which stabilizes the material's initial resistance value. When target gas molecules contact the surface of the material, they interact with the adsorbed oxygen species. This leads to a decrease or transformation of the surface oxygen species, thereby breaking the initial surface state equilibrium and causing changes in the material's resistance. This is also the core principle behind the gas sensing capabilities of semiconductor aerogels [164]. For example, Liu et al. [91] constructed a Bi_2_S_3_/MoS_2_ nanocomposite aerogel. In ambient air at room temperature, oxygen molecules preferentially adsorb onto the MoS_2_ surface and heterojunction interface. This captures free electrons through chemical adsorption, ionizing the oxygen O_2_^−^. This process is accompanied by electron transfer from Bi_2_S_3_ to MoS_2_, promoting the thickening of the electron depletion layer and establishing a stable initial resistance for the sensing material. The adsorbed O_2_^−^, as an active species, subsequently reacts with NO_2_ to generate NO_3_^−^, significantly amplifying the resistance change signal (Figure 11b). The sensor exhibits a response value of 49.58 to 50 ppm NO_2_, which is approximately 15.3 times greater than that of pure MoS_2_, and has fast response and recovery times of 3.2 and 56.5 s, respectively.

#### 3.2.2 Charge Transfer Mechanism

The charge transfer mechanism is a fundamental gas sensing process that is distinct from the adsorbed oxygen ion mechanism. The latter relies directly on the charge transfer process between gas molecules and gas gel sensing materials to produce a sensing response.

In the core logic of the sensing process, aerogel sensing materials primarily act as charge receptors or donors within the reaction system. When target gas molecules diffuse to the surface of the material and undergo adsorption due to differences in their electronic structures and that of the sensing material, a spontaneous, directional, and quantitative charge transfer occurs between them [165]. This directly alters the carrier concentration of the aerogel material, inducing significant changes in its resistance. Furthermore, the physicochemical properties of different gas molecules can result in different amounts and directions of charge transfer, which ultimately manifests as specific differences in the trend and magnitude of material resistance changes [166]. For example, the 3D Ag‐rGO composite aerogel prepared by Li et al. [167] through a one‐step hydrothermal method is a typical representative of this mechanism. When the sensing material is exposed to air, oxygen molecules undergo chemical adsorption on the material surface and capture electrons to form oxygen ions (Equation 5). When exposed to NO_2_, NO_2_ molecules with high electron affinity directly act as strong electron acceptors, capturing electrons from the Ag‐rGO material (Equation 8). This direct transfer is the fundamental cause of resistance changes. Simultaneously, the adsorbed oxygen ions (O_2_^−^) can react with NO_2_ to generate NO_3_^−^ (Equation 9), which can consume more electrons synergistically, thereby amplifying the changes in carrier concentration induced by direct charge transfer. In other words, the high‐affinity layer (HAL) is significantly broadened, and the high‐density layer (HDL) is narrowed, collectively leading to a sharp decrease in material resistance (Figure 11c).

(8) $$NO_{2}\left(\mathrm{gas}\right)+e^{-}\to N{O_{2}}^{-}\left(\mathrm{ads}\right)$$

(9) $$2NO_{2}\left(\mathrm{gas}\right)+e^{-}+{O_{2}}^{-}\left(\mathrm{ads}\right)\to 2N{O_{3}}^{-}\left(\mathrm{ads}\right)$$

The sensing process also exhibits good reversibility. When the aerogel sensing material is re‐exposed to the air environment, the adsorbed gas molecules will desorb due to changes in the ambient gas composition. The charge balance state on the material surface gradually recovers, and the carrier concentration returns to its initial level. Correspondingly, material resistance also recovers to its initial value. This reversibility enables the sensor to be used repeatedly [168].

#### 3.2.3 Equivalent Circuit Mechanism

The equivalent circuit mechanism, based on the core‐shell structure of semiconductor grains, provides an explanation of the essence of the gas sensing response through a simplified circuit model. The core concept utilizes the difference in resistance between the grain surface and the core to reveal the correlation between gas adsorption and resistance changes. This approach is applicable to most semiconductor gas sensing materials [169, 170].

**FIGURE 12:**
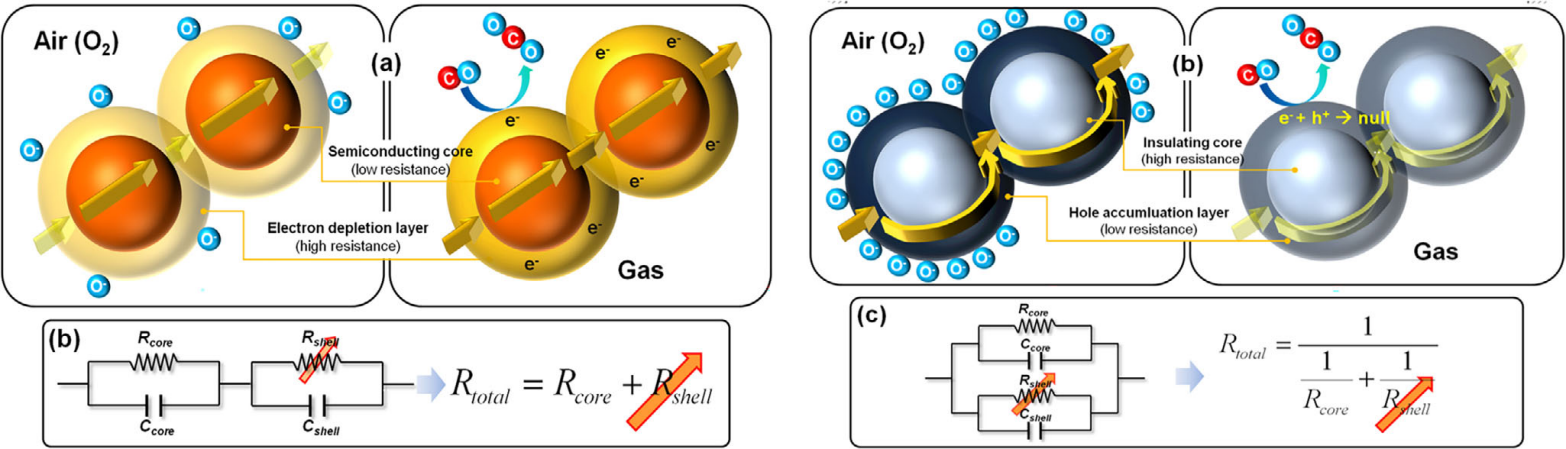
n‐type and p‐type oxide semiconductor core‐shell structures and equivalent circuit simulation diagrams. Reproduced with permission [171]. Copyright 2014 Elsevier.

Oxygen in the air captures electrons from the surface of sensitive materials, forming a core‐shell structure in the grains. A high‐resistance electron depletion layer (shell layer) forms on the surface of n‐type semiconductors, with the core being a low‐resistance region. The total resistance is equivalent to a series circuit of the shell contact resistance (R _shell_) and the core resistance (R _core_), with R _core_ being the dominant factor. Conversely, a low‐resistance hole accumulation layer (shell layer) is formed on the surface of p‐type semiconductors, with the core being a high‐resistance region. The total resistance is equivalent to a parallel circuit of R _shell_ and R _core_, with conductivity concentrated in the shell region (Figure 12) [171].

During gas detection, when an n‐type semiconductor encounters a reducing gas, it absorbs oxygen and reacts with the gas to release electrons. This narrows the depletion layer, reduces the R _shell_ resistance, and lowers the total resistance. Conversely, when exposed to oxidizing gas, the resistance increases. The behavior of a p‐type semiconductor is opposite: upon encountering a reducing gas, the reaction releases electrons that recombine with holes, decreasing the hole concentration. This increases the shell resistance and raises the total resistance. For example, Sil et al. [172] studied the capacitive and resistive gas sensing characteristics of ZnO homojunctions, and their gas sensing mechanism aligns with the equivalent circuit mechanism. In the presence of O_2_ in the air, n‐type ZnO nanotubes and p‐type ZnO nanoparticles form a core‐shell structure, corresponding to different equivalent circuits. The n‐type semiconductor is represented as a series connection of R _shell_ and R _core_, while the p‐type is represented as a parallel connection. When exposed to reducing gases such as methanol, the depletion layer of the n‐type semiconductor narrows, and the R _shell_ decreases. The change in the p‐type hole accumulation layer then leads to an increase in shell resistance, thereby achieving a gas sensing response through changes in the equivalent circuit parameters. In capacitive sensing, the resistance and capacitance components of the equivalent circuit work together, achieving a methanol response of 3021% and a significantly superior selectivity window to that of resistive sensing. This confirms the explanatory power of the equivalent circuit mechanism for semiconductor gas sensing characteristics. A quantitative relationship exists between the gas sensing responses of p‐type and n‐type semiconductors (Equation 10) [173]:

(10) $$\mathrm{Sp}=\sqrt{Sn}$$

where Sp represents the response value of p‐type sensing material, and Sn represents the response value of n‐type sensing material.

#### 3.2.4 Electron Scattering Mechanism

When the particle size (thickness) of the gas‐sensitive material is tuned to be on the same order of magnitude as the mean free path of carriers, the transport process of carriers within the material will exhibit strong sensitivity to surface states. At this point, gas molecules adsorbed on the material's surface become significant centers for scattering electrons, interfering with the directional migration of carriers through Coulomb interactions or lattice distortion effects and effectively hindering the conduction path of free charge carriers. This scattering effect is highly efficient in regulating the process, and even the adsorption of a small number of gas molecules can trigger a sharp decrease in carrier mobility, leading to significant attenuation of the material's conductivity.

The sensing mechanism, which is dominated by electron scattering, overcomes the strong dependence of traditional gas‐sensing materials on the number of active sites on the surface. Its sensing response is based on the perturbation of carrier transport paths rather than on simple surface charge transfer. This makes the material more tolerant of surface modification and improves response stability. Furthermore, this mechanism is fundamental to room‐temperature gas sensing, as the average free path of carriers decreases with increasing temperature, thereby weakening the regulatory effect of scattering centers in high‐temperature environments. At room temperature, however, the match between the average free path of the carriers and the size of the material particles (thickness) is better, enabling the full utilization of the scattering effect. This ensures a highly sensitive response and avoids issues such as high energy consumption and decreased material stability associated with high‐temperature sensing. Liu et al. [174] designed a room‐temperature NO_2_ sensing material based on a mesoporous In_6_WO_12_/Pr_2_O_3_ heterojunction. The material's crystal grain size of the material (8–10 nm) is comparable to the average free path of carriers at room temperature. Following NO_2_ adsorption, the material becomes a scattering center, hindering electron migration and significantly increasing resistance, thereby enhancing the sensing signal. Additionally, the crystal grain size is smaller than the Debye length (approximately 21 nm), enabling almost all electrons to participate in sensing via spatial charge transfer NO_2_ captures electrons, further increasing the resistance change. Furthermore, the loading of Pr_2_O_3_ reduces the material's work function and increases the content of chemisorbed oxygen (O_2_^−^). O_2_^−^ reacts with NO_2_ to generate NO_3_^−^, which cooperatively captures electrons.

## 4 Sensing Applications of Aerogel in Different Types of Gases

### 4.1 Nitrogen Oxides

NO_x_ is a common secondary atmospheric pollutant, posing a serious threat to the stability of the ecological environment and to human health. The main sources of its emission include vehicle exhaust fumes, thermal power generation, industrial coal combustion, metallurgy, and other high‐temperature combustion processes. Additionally, small amounts of emissions are produced by biomass combustion and some chemical processes [175, 176]. In the atmosphere, NO_x_ can participate in photochemical reactions, combining with volatile organic compounds, carbon monoxide, and other pollutants to generate ozone and other strong oxidizing agents, causing photochemical smog. It can also promote the secondary formation and accumulation of fine particulate matter by converting into nitrate aerosols, which significantly exacerbates haze pollution [177, 178]. Furthermore, long‐term exposure to NO_x_ can damage the respiratory and cardiovascular systems in humans. This multifaceted hazard makes the development of efficient, sensitive NO_x_ detection technology urgent in the field of environmental monitoring [179].

Currently, strategies for improving the sensing performance of NO_2_ at room temperature focus on optimizing the electronic structure of aerogels, creating efficient interfaces, and controlling porous networks. Integrating multifunctional components to combine their advantages can significantly improve performance. Yuan et al. [180] synthesized ZnO/rGO composite aerogels using ZIF‐8/GO as a precursor. In this system, the rGO aerogel forms a highly conductive 3D network, and its numerous sp^3^ defects increase its interaction with NO_2_. This composite material exhibits high sensitivity to NO_2_ at room temperature (100 ppm NO_2_ 3.21), with a response time of 268 s, and its porous structure facilitates gas transmission and reaction, demonstrating its potential for use in monitoring factory and atmospheric environments. Chen et al. [181] prepared a 3D Pd‐In_2_O_3_/rGO aerogel via a one‐step hydrothermal synthesis and freeze‐drying process. The Pd‐In_2_O_3_ nanoparticles are uniformly grown on the rGO thin layer. The aerogel's high specific surface area and abundant porous structure effectively inhibit the agglomeration and accumulation of nanoparticles, promoting the formation of p‐n heterojunctions and ultimately achieving efficient H_2_ detection at room temperature. The sensitivity to 10 000 ppm H_2_ is 27.66, demonstrating stable detection performance and good reproducibility. The response and recovery times are as short as 11 and 13 s, respectively, providing a reference for optimizing multi‐gas sensing systems.

**FIGURE 13:**
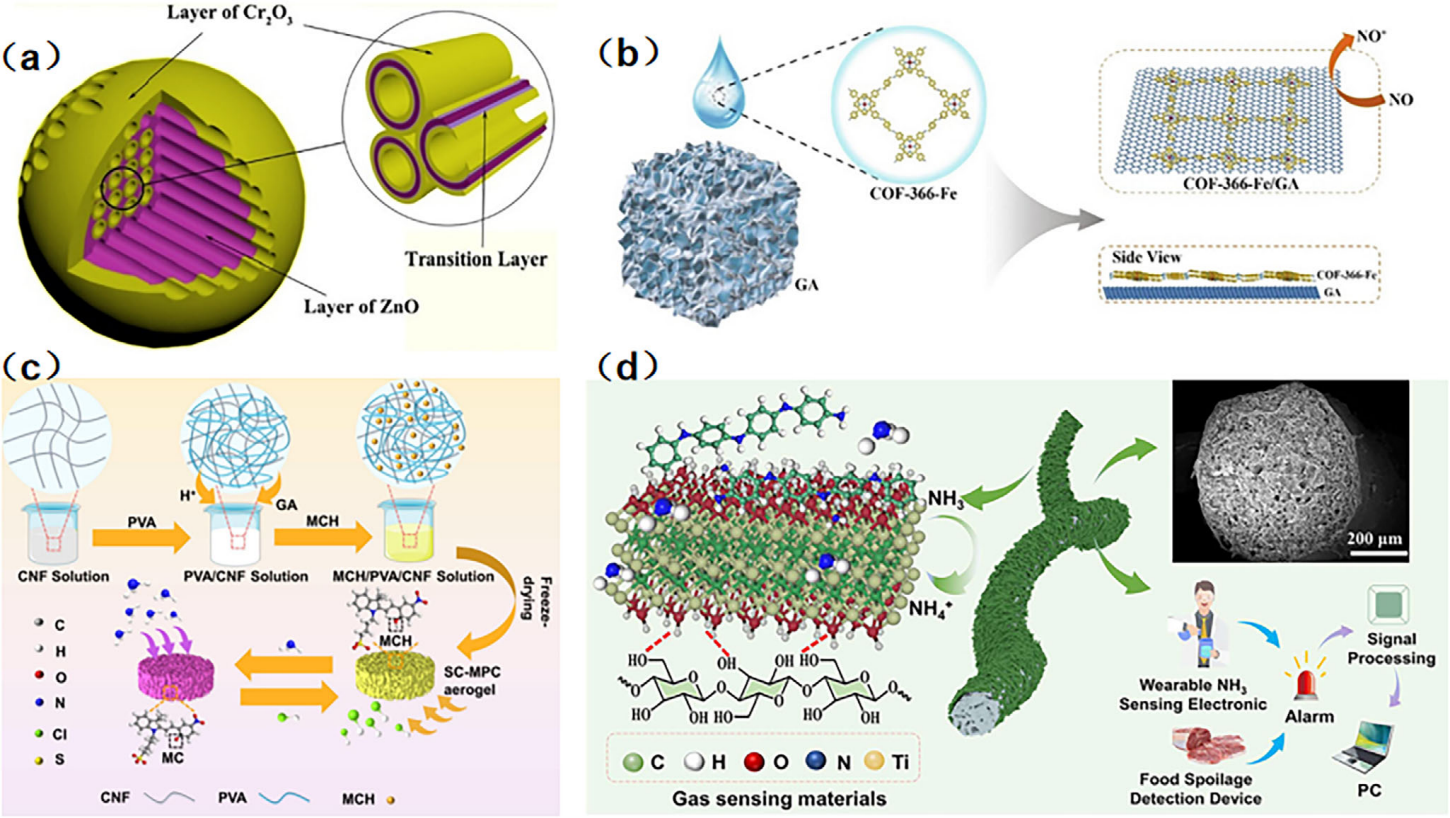
(a) Schematic diagram of the microstructure of the prepared OP‐Cr_2_O_3_/ZnO sample. Reproduced with permission [182]. Copyright 2018 Elsevier. (b) Schematic diagram of COF‐366‐Fe/GA as the sensing component for identifying NO molecules. Reproduced with permission [17]. Copyright 2021 Elsevier. (c) Flowchart showing the preparation and color change mechanism of SC‐MPC aerogel. Reproduced with permission [195]. Copyright 2025 Elsevier. (d) Schematic diagram of BA/MXene/PANI aerogel fibers and SEM image of the cross‐section of BA/MXene/PANI aerogel fibers. Reproduced with permission [196]. Copyright 2025 American Chemical Society.

In addition to the above composite of multiple materials, the construction of heterojunctions is also a key approach to enhancing sensing performance. For instance, Wang et al. [182] produced an ordered, porous Cr_2_O_3_/ZnO aerogel featuring a p‐n heterojunction structure via ultrasonic chemical deposition. The aerogel's unique ordered porous structure provides ideal channels for gas diffusion, while the p‐n heterojunction formed between the two materials effectively promotes the separation and transfer of interface charges (see Figure 13a for the sensing mechanism). The synergistic effect of the two components enables the sensing material to achieve a high response of 86.7% to 100 ppm NO_2_ at room temperature (25 ± 2°C), with a response time of only 4 s and a detection limit of 0.05 ppm. It also exhibits excellent selectivity. Yin et al. [183] synthesized MoS_2_‐based nanocomposites using a two‐step growth method involving activation of the substrate surface of the constructed material. The N‐doped graphene and MoS_2_ nanocomposites form a large heterojunction interface. The aerogel exhibits a high response of 24.3% to 50 ppm NO_2_ at 25°C, demonstrating high selectivity, long‐term stability, and a ppb‐level detection limit. Xing et al. [184] successfully synthesized a nano‐flower‐like γ‐In_2_Se_3_/In_2_O_3_ heterojunction via a solvothermal process followed by air thermal oxidation. The resistive sensing material constructed based on this structure exhibits significantly enhanced response characteristics to NO_2_ at an operating temperature of 170°C. The sensor's response value to 0.2 ppm NO_2_ can reach 2.69, and to 8 ppm NO_2_, it can reach 9.68. Research shows that the nano‐flower structure increases the material's specific surface area (20.851 m^2^/g) and surface‐active sites, thereby enhancing its sensitivity to NO_2_. Meanwhile, the heterojunction formed between γ‐In_2_Se_3_ and In_2_O_3_ promotes charge transfer between NO_2_ molecules and the sensing material, further enhancing the response signal. In this study, the nano‐flower‐like γ‐In_2_Se_3_/In_2_O_3_ material was obtained by in situ air oxidation at high temperatures of pre‐synthesized nano‐flower‐like γ‐In_2_Se_3_. The gas sensor based on this heterojunction exhibits outstanding sensitivity.

Through the synergistic integration of functional material composites and innovative sensing mechanisms, aerogel sensing materials can achieve the efficient and precise detection of NO_2_ in the environment, as well as expand into sensing applications related to human physiology. This overcomes the limitations of traditional sensing materials. Zhu et al. [17] created a COF material on the surface of a 3D graphene aerogel (GA) via in situ growth to produce a COF‐based aerogel (Figure 13b). This material cleverly combines the excellent electrocatalytic activity of COF materials with the 3D porous structure of graphene aerogel. Room temperature sensing performance tests revealed that the COF‐based aerogel modified electrode exhibits an ultra‐wide linear response range for NO_2_ (0.18–400 µM), with a sensitivity of up to 8.8 µA·µm^−1^·cm^−1^, a detection limit of 30 Nm, and a response time of less than 3 s. This performance far surpasses that of traditional metal‐semiconductor sensing materials. It also exhibits excellent selectivity toward common interfering substances, such as calcium nitrate, glucose, and dopamine, with response ratios exceeding 9.6 for all of them. It also possesses good long‐term stability and biocompatibility. This material can successfully monitor NO secretion by human umbilical vein endothelial cells in real time, demonstrating its unique value in the research of physiological and pathological processes, as well as in clinical diagnosis. Furthermore, Nufer et al. [185] reported a one‐step process for synthesizing carbon aerogels through laser deposition technology, producing a porous, conductive film composed of carbon clusters directly. This aerogel exhibits high selectivity toward nitrogen dioxide (NO_2_) and can detect concentrations as low as one part per billion (ppb). Notably, its sensing mechanism is based on the dissolution of NO_2_ in the water layer adsorbed onto the hydrophilic surface of the aerogel, which enhances the usability of the sensing material in environments with variable humidity.

The NO_2_ sensing performance at room temperature can also be effectively improved by adjusting the material defects and crystal structure to optimize the electronic transport characteristics and gas adsorption capacity of the aerogel. Jyoti et al. [186] synthesized CNT aerogel films via floating catalyst chemical vapor deposition. These films comprise a fine network of elongated CNTs and approximately 20% amorphous carbon. Following heat treatment at 700°C, the material's pore structure and defect density were precisely controlled. The resulting sensor film demonstrated outstanding sensitivity toward NO_2_ and methanol gas across a concentration range of 1–100 ppm, with a detection limit as low as approximately 90 ppb. It maintained a stable response to toxic gases even after bending and crumpling. In contrast, films heat‐treated at 900°C exhibit reduced response values and inverted sensing characteristics due to the CNT aerogel's semiconductor nature shifting from p‐type to n‐type. This provides novel insights for optimizing sensing performance through temperature regulation.

### 4.2 Ammonia Gas

Ammonia (NH_3_) is an important chemical raw material that is widely used in fields such as the production of nitrogen fertilizers and industrial refrigeration [187, 188]. However, the issue of ′ammonia escape″ associated with its transportation and use has not received sufficient attention for a long time. This not only exacerbates atmospheric haze pollution but also directly impacts human health. High concentrations of ammonia can cause serious respiratory diseases, as well as damage the heart, eyes, and skin [189, 190]. Conversely, ammonia is a natural by‐product of human metabolism, and its concentration in exhaled breath is closely related to liver or kidney disease. Therefore, related diseases can be screened early on by sensing the ammonia content in exhaled breath [191, 192]. In this context, developing real‐time, high‐sensitivity ammonia detection technology suitable for different scenarios is highly significant [193, 194].

The colorimetric sensing method is a widely used method for sensing NH_3_, but it often encounters issues such as limited sensitivity, difficulty in achieving accurate quantification, and inadequate long‐term stability. For instance, the sulfonated spirocyclopyridinyl intelligent colorimetric aerogel (SC MPC aerogel), developed by Liu et al. [195], exhibits a significant color change upon contact with ammonia (Figure 13c). However, the quantitative ability and detection limit of such methods often fail to meet the requirements of high‐precision environmental monitoring or medical diagnosis.

In view of the limitations of the colorimetric sensing method for high‐precision ammonia detection, the resistance‐type aerogel sensing method has successfully overcome the above technical bottlenecks through material composition optimization and a unique structural design, achieving ultra‐sensitive ammonia detection. Huang et al. [196] developed a fully biodegradable flexible fibrous ammonia sensing material. Utilizing MXene/PANI doped with organic sulfonic acid (PAMPS) as the sensing layer (Figure 13d), it exhibited a response value as high as 807% at 100 ppm NH_3_. Remarkably, it still achieved a 19% response even at an ultra‐low concentration of 1 ppb NH_3_. This material simultaneously demonstrated rapid response/ recovery characteristics (24.1 s/2.2 s) and excellent selectivity. Crucially, this material can be completely degraded within 10 days in a 2 wt.% H_2_O_2_ solution, meeting green environmental requirements. Li et al. [124] synthesized an LCNF/ZnO composite aerogel for ammonia gas sensing using a hydrothermal reaction combined with freeze‐drying technology. Introducing a small amount of CNT to optimize the electronic conduction path significantly improved the room temperature sensing performance. The ZnO response value to 50 ppm of ammonia at room temperature is 4.94%, with a recovery time of only 13.1 s, and the detection limit is as low as 5 ppb. Furthermore, the material is highly degradable. It can be completely degraded within 3 weeks when buried in soil. This not only expands the application of ZnO in the real‐time monitoring of room‐temperature NH_3_ but also provides new ideas for the green and sustainable utilization of LCNFs. Fernando et al. [197] successfully synthesized a SnO_2_‐polystyrene sulfonate (PEDOT: PSS) composite aerogel material using the epoxy resin method. All SnO_2_ ‐ based aerogels demonstrate high selectivity for ammonia, with a detection limit for hybrid aerogel materials of up to 50 ppb. Notably, the SnO_2_/PANI composite material achieved an ultra‐low ammonia detection limit of 2 ppb. At a concentration of 20 ppm of ammonia, the response values of the SnO_2_ sensor and the SnO_2_/PEDOT: PSS sensors were 73.7% and 91.4%, respectively.

In addition, composite aerogels based on carbon materials also demonstrate excellent sensing capabilities. For instance, Li et al. [198] synthesized boron nitride‐coated graphene aerogel (BN‐GA) using graphene aerogel as a template. Leveraging the gas selectivity regulation of the h‐BN coating layer, they achieved ultra‐high‐sensitivity detection of NH_3_ with a detection limit as low as the ppb level. The detection range spanned 20–10 ppm, demonstrating excellent selectivity toward other common gases. Kuang et al. [199] employed rGO aerogel as a template to synthesize silicon carbide/rGO (SiC/rGO) nanosheets via high‐temperature gas‐solid reaction, utilizing them for selective NH_3_ sensing at ambient temperatures. This material inherits the layered structure of rGO, forming abundant gas‐sensitive active sites during the GO reduction process. The sample synthesized at 1500°C and annealed at 900°C in air (SiC‐1500‐900) exhibited optimal performance. At room temperature and 30% relative humidity, the quartz crystal microbalance (QCM) sensor constructed from this material exhibited a frequency shift of 75.98 Hz in response to 50 ppm NH_3_, with response/recovery times of 16.2 s/19.4 s, respectively. This response value was 8.5 times that of the unannealed sample. This enhancement stems from the synergistic interaction between the nanosheet structure and the hydroxyl groups introduced by annealing, which promote affinity reactions with ammonia molecules. This confirms that controlled oxidation serves as an effective strategy for optimizing the surface hydrophilicity and sensing performance of SiC/rGO nanosheets.

### 4.3 Aldehydes

Volatile organic compounds (VOCs), as complex and particularly harmful pollutants in the atmosphere, require efficient detection to improve air quality and safeguard human health [200, 201]. Aldehyde VOCs, such as formaldehyde and acetaldehyde, are among the main sources of indoor air pollution. These substances are strong irritants and are toxic, long‐term exposure can damage the respiratory system and organ function. Their synergistic effect has also been proven to significantly increase the risk of cancer [202]. Therefore, developing technologies that can detect aldehyde VOCs with high sensitivity and selectivity in real time is of great significance.

**FIGURE 14:**
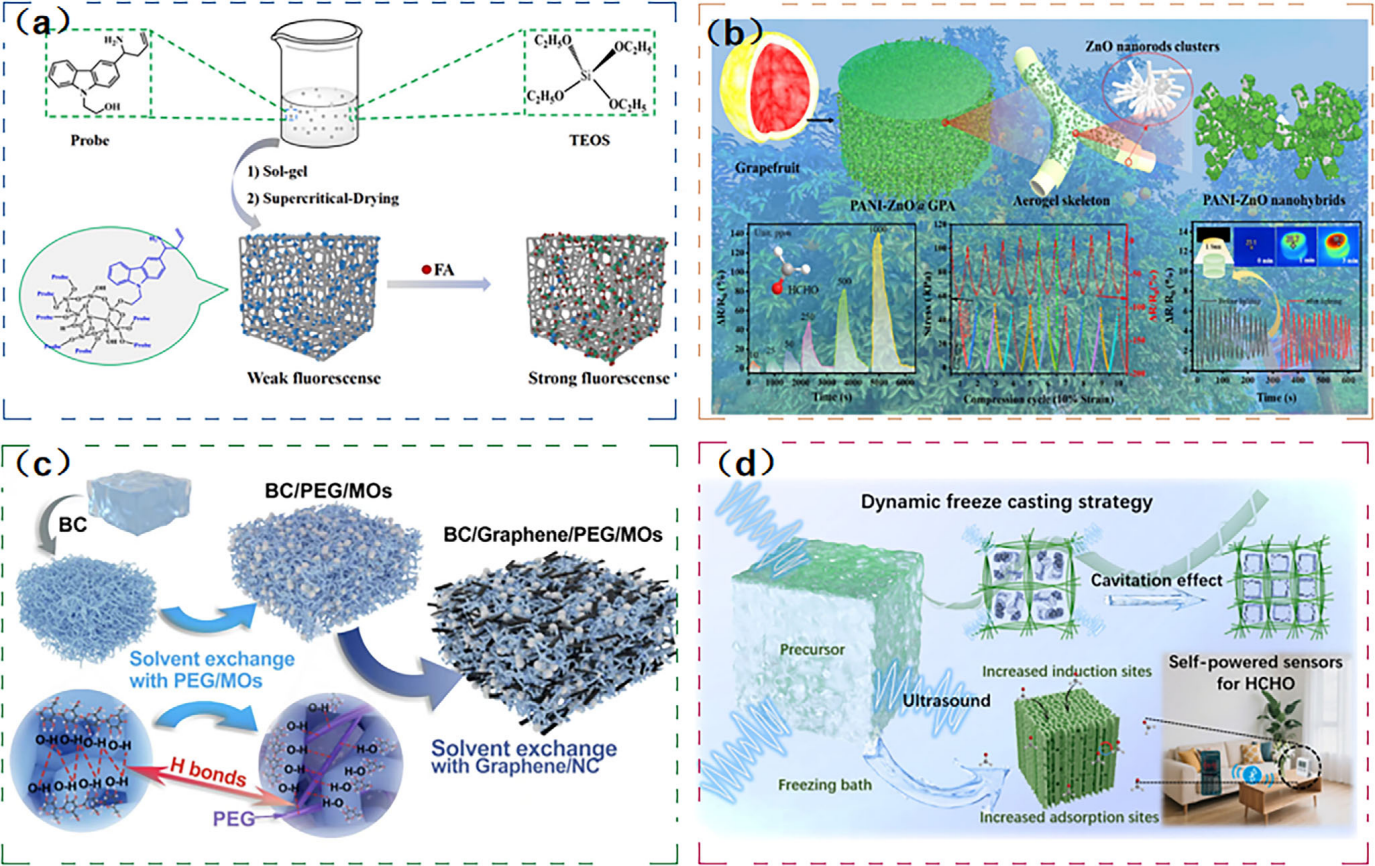
(a) Synthesis route of Probe‐SiO_2_ fluorescent probe aerogel. Reproduced with permission [203]. Copyright 2021 Elsevier. (b) Structure and performance testing of PANI‐ZnO@GPA aerogel. Reproduced with permission [204]. Copyright 2022 American Chemical Society. (c) Microstructure of BC/PEG aerogel. Reproduced with permission [205]. Copyright 2024 Elsevier. (d) Preparation and schematic diagram of cellulose triboelectric aerogel. Reproduced with permission [206]. Copyright 2025 American Chemical Society.

The fluorescence detection method is a traditional method of aldehyde detection. For instance, Zheng et al. [203] reported a new sensing material (Figure 14a) consisting of a silica‐based aerogel skeleton onto which a specific fluorescent probe molecule had been covalently grafted. This design combines the gas adsorption characteristics of the aerogel with the sensitivity and selectivity of the molecular probe. When formaldehyde gas diffuses into the pores of the aerogel and reacts specifically with the probe, formaldehyde can be detected selectively. However, the optical sensing strategy has obvious shortcomings, and the detection limit of fluorescence sensing is generally high. This makes it difficult to meet the precise monitoring needs of low‐concentration aldehyde VOCs. These shortcomings have prompted researchers to turn their attention to sensing paths that are more suitable for practical applications.

To address these issues, researchers have suggested various methods, one of which is the composite aerogel resistance sensing strategy based on heterostructure interface construction and optimization of the conductive network. Wang et al. [204] successfully constructed the PANI‐ZnO@GPA composite aerogel. The p‐n heterojunction formed between the PANI and ZnO promotes interface charge separation and transfer, significantly enhancing the electrical signal response. The sensing material exhibits sensitivity of up to 0.134% ppm^−^^1^ to formaldehyde within a concentration range of 10–1000 ppm at room temperature, with a detection limit as low as 2.32 ppm (Figure 14b). Wei et al. [205]used the solution displacement method to load metal oxides and graphene nanosheets onto polyethylene glycol‐modified BC aerogels (Figure 14c). The resulting composite material makes full use of the high permeability and abundant pores of the 3D BC nanofiber network to expand the contact area between the MOS/graphene and VOC molecules, thereby achieving excellent sensing performance. The detection limits for formaldehyde and acetone are 0.75 and 1.43 ppm, respectively. Wang et al. [206] proposed a dynamic freeze‐casting strategy based on the cavitation effect, successfully preparing triboelectric aerogels with tunable pore sizes and significantly enhanced sensitivity (Figure 14d). The porous structure of this material not only increases the induced charge density but also provides abundant reaction sites for formaldehyde molecules. Test results show that the sensor based on this aerogel has a response time of only 6.6 s for formaldehyde, a full recovery time of 10.5 s, and a minimum detection limit of 0.035 mg/m^3^.

### 4.4 Structure‐Property Relationships and Application Challenges of Aerogels

The gas‐sensing performance of aerogels depends not only on material composition but is also closely related to their microstructure. High specific surface area, hierarchical pore structures, and heterojunction interfaces collectively influence gas adsorption, diffusion, mass transfer, and electron transport processes, thereby determining the sensor's sensitivity, response recovery rate, and selectivity. Therefore, a thorough understanding of the relationship between structural parameters and sensing performance is crucial for the rational design of high‐performance gas‐sensing aerogels. To provide a more intuitive analysis of the relationship between aerogel structural parameters and sensing performance, Table 1 summarizes the specific surface area, pore structure characteristics, and heterojunction types of representative gas‐sensitive aerogels, along with their corresponding sensing performance and engineering performance.

**TABLE 1:** Relationship between structural parameters and sensing performance of aerogel gas sensors.

Material	Specific surface area (m^2^/g)	Aperture (nm)	Heterojunction type	Gas	Detection limit	Response/recovery time (s)	Stability (day)	Cycles	Moisture resistance (%RH)	Ref.
MXene/rGO/CuO	—	—	p‐p	C_3_H_6_O	10ppm	6.5/7.5	—	3	20–60	[76]
Pd/TiO_2_	172.55	—	Pd‐ n	H_2_	100 ppm	<2/<22	—	30	30	[79]
Bi_2_S_3_/MoS_2_	16–21	—	p‐n	NO_2_	50 ppm	3.2/56.5	10	70	—	[91]
Pt/CNT	73.87	—	Pt‐CNT	H_2_	3000 ppm	31/4	15	6	20–98	[123]
CuM/MXene	482	5.3	CuM/MXene	NO_2_	5 ppb	0.9/4.5	56	5	22.8–23.2	[133]
MXene/PANI/BC	—	—	MXene‐PANI	NH_3_	56.49 ppb	—/—	7	5	30–80	[141]
Cr_2_O_3_/ZnO	46.1	5.48	p‐n	NO_x_	0.05 ppm	9/—	—	>180	—	[182]
In_2_O_3_/SnO_2_	376.095	2.39	p‐n	NO_x_	0.08 ppm	14/—	>180	—	20–80	[210]
T‐Ni‐HHTP/C‐CNT	1078.62	17.9	p/CNT	NO	3 ppb	4/9	7	120	10–90	[212]
rGO/Eu (TPyP)(Pc)	96.4	5.6	p‐n	NO_2_	100 ppm	172/828	120	—	30–100	[213]
SnO_2_/rGO	—	—	p‐n	HCHO	8.02 ppb	—/—	50	50	—	[217]

**FIGURE 15:**
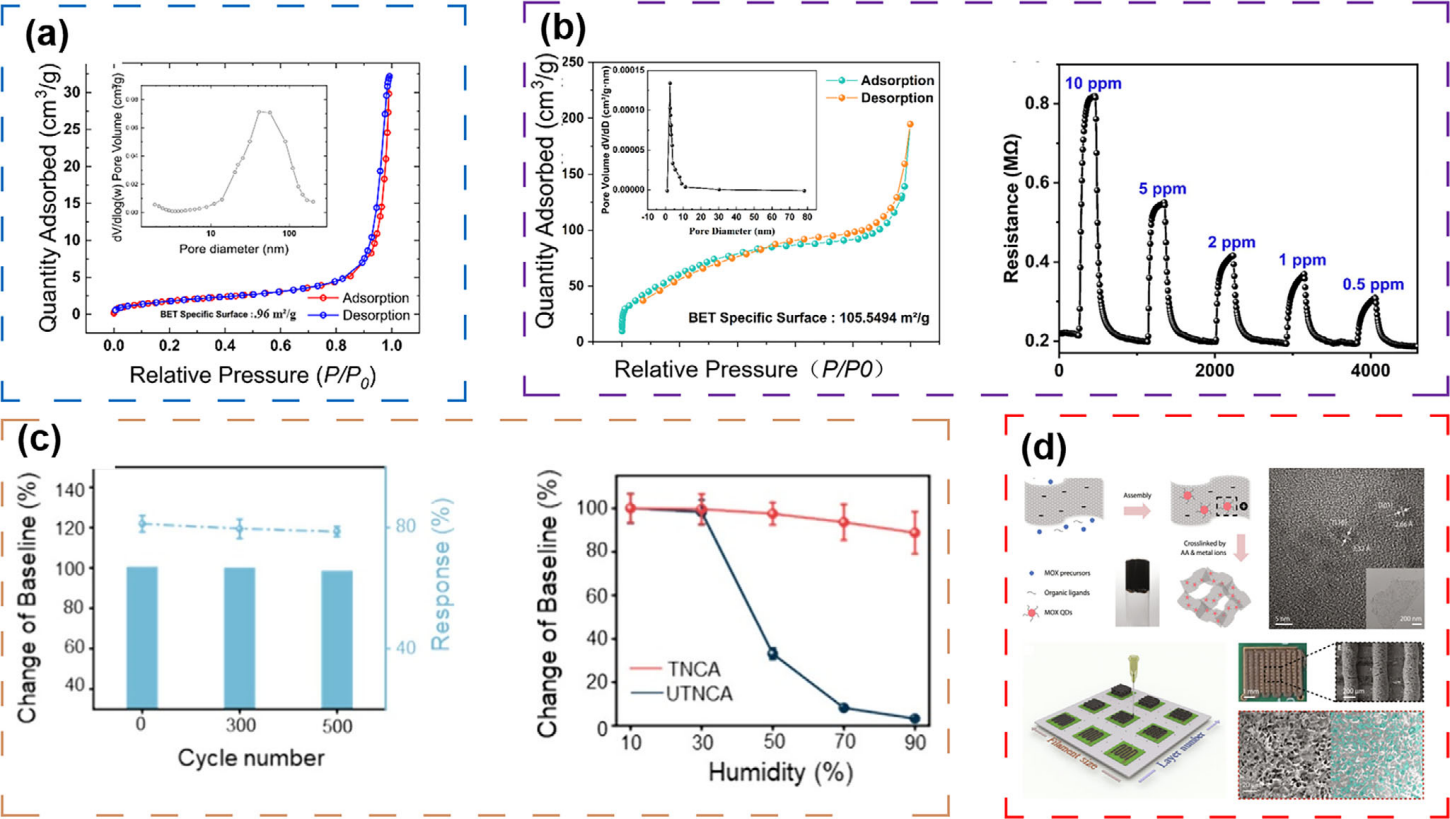
(a) Specific surface area of WO_3_ particles. Reproduced with permission [207]. Copyright 2025, Elsevier. (b) Specific surface area of WO_3_ aerogel. and Relationship between the resistance of the aerogel sensor and nitrogen dioxide concentrations ranging from 0.5 to 10 ppm. Reproduced with permission [208]. Copyright 2026, Elsevier. (c) after 300 cycles, and after 500 cycles. and Percentage of baseline resistance at different humidity levels. Reproduced with permission [212]. Copyright 2026 American Chemical Society. (d) Hybrid material ink formulation and 3D printed aerogels. Reproduced with permission [217]. Copyright 2024, American Association for the Advancement of Science.

Specific surface area is a key factor influencing gas adsorption capacity. A larger specific surface area provides more active sites and promotes the uniform dispersion of active components, thereby enhancing gas adsorption and surface reactions. For example, when WO_3_ was transformed from a traditional particulate structure into a three‐dimensional mesoporous aerogel, its specific surface area increased to 105.5 m^2^/g, and the detection limit for NO_2_ was reduced to 0.5 ppm, nitrogen‐doped activated carbon/graphene composite aerogel has a specific surface area of 621.2 m^2^/g, and its response sensitivity to formaldehyde is 4.2 times higher than that of pure graphene aerogel [113, 207, 208] (Figure 15a,b). These results indicate that a high specific surface area increases the probability of contact between gas molecules and active sites, thereby enhancing the sensing response.

In addition to the number of active sites, the pore structure also determines the efficiency of gas diffusion within the material. An optimal pore size distribution can balance both the gas diffusion rate and the utilization of active sites, thereby achieving a combination of high sensitivity and fast response. Studies have shown that for common target gases such as NO_x_, NH_3_, and aldehydes, a mesoporous structure with pore sizes ranging from 2 to 50 nm is more conducive to balancing diffusion and reaction processes [209]. The CuO/ZnO aerogel prepared by Jaihindh et al. has an average pore diameter of approximately 31.8 nm and open, interconnected channels, which significantly reduce gas diffusion resistance and thus exhibit excellent response and recovery performance [46]. Therefore, optimizing the pore structure not only affects mass transfer efficiency but is also a key approach to regulating dynamic response behavior.

Heterojunction interfaces can further enhance electron transport and signal amplification. The built‐in electric field and band bending formed when different materials come into contact facilitate carrier separation and migration, thereby amplifying the electrical signal changes caused by gas adsorption. For example, the Bi_2_S_3_/MoS_2_ p–n heterojunction increases the response to 50 ppm NO_2_ to more than 15 times that of pure MoS_2_, while the In_2_O_3_/SnO_2_ and rGO/MXene heterostructures similarly improve gas detection performance through interfacial charge redistribution [91, 210, 211]. Thus, a high specific surface area provides ample reaction sites, an optimized pore structure ensures efficient mass transfer, and heterojunction interfaces enable signal amplification, together, these three factors constitute the core framework of the aerogel structure–performance relationship.

It is worth noting that there is currently a lack of a unified quantitative structure–property relationship regarding the specific surface area, pore size, and heterojunction structure in relation to sensing performance. Response values do not always increase linearly with increasing specific surface area, the optimal pore size range also varies depending on the target gas type and material system, and an increase in the number of heterojunctions does not necessarily lead to continuous performance enhancement. Therefore, the design of most gas‐sensitive aerogels currently relies primarily on empirical optimization. In the future, there is an urgent need to establish quantitative predictive models linking structural parameters to sensing performance by integrating in situ characterization, multiscale simulations, and machine learning‐aided design.

Although structural design can significantly enhance sensing performance, high sensitivity does not necessarily translate to excellent practical applicability. In complex environments and under long‐term service conditions, aerogel sensors still face engineering challenges such as long‐term stability, humidity interference, and large‐scale manufacturing.

Long‐term stability and repeatability are key indicators for evaluating the practical value of gas sensors. The T‐Ni‐HHTP/C‐CNT heterostructured MOF aerogel developed by Zhao et al. exhibited response curves that were essentially identical across seven consecutive NO detection cycles, maintained stable detection performance after 120 days of storage, and retained stable baseline resistance and response signals after 500 compression cycles [212] (Figure 15c). After 500 mechanical cycles under 70% RH conditions, the sensor retained 83.6% of its response intensity, indicating that the stable three‐dimensional porous framework effectively maintains the gas diffusion channels and electronic transport networks, thereby ensuring long‐term detection reliability.

Ambient humidity is a key factor affecting the practical application of aerogel sensors. Since water molecules readily compete with target gases for adsorption sites and alter the surface charge distribution, this often leads to response attenuation and baseline drift. Currently, researchers primarily enhance moisture resistance through the construction of hydrophobic interfaces and the design of heterostructures. Zhao et al. used POTS to hydrophobically modify aerogels, within a range of 10%–90% RH, the change in the sensor's baseline resistance was only 11.3%, significantly lower than the 96.2% observed in untreated samples, and the sensor maintained a stable response even after 180 min of continuous operation at 90% RH [212] (Figure 15c). Furthermore, Zhu et al. effectively suppressed water molecule adsorption on aerogels by utilizing rough surfaces formed by low‐surface‐energy interfaces and three‐dimensional porous frameworks, thereby maintaining stable NO_2_ detection performance even in high‐humidity environments [213].

In addition to environmental adaptability, the capacity for large‐scale manufacturing is also a crucial prerequisite for advancing the practical application of aerogels. Pan et al. [214] prepared a bio‐based aerogel with an optimized pore structure by modulating the gel network structure with PVA and combining it with freeze‐drying. This aerogel not only achieved sensitive detection of 10 ppm NH_3_ but was also successfully applied to smart labels for food freshness, demonstrating the application potential of gas‐sensitive aerogels in real‐world scenarios. However, traditional freeze‐drying and supercritical drying processes generally suffer from high energy consumption, long processing cycles, and high equipment costs, which limit their industrial‐scale adoption.

To reduce manufacturing costs and improve production efficiency, researchers have begun exploring continuous and low‐energy‐consumption preparation technologies. Jiang et al. [215] employed a combination of coaxial wet spinning and atmospheric‐pressure rapid drying to achieve the continuous preparation of cellulose composite aerogel fibers, providing a new approach for low‐cost, large‐scale production. At the same time, advanced manufacturing technologies such as 3D printing have also opened new technical pathways for the precise fabrication and mass production of aerogels with complex structures. Yang et al. [216] proposed an additive‐free aerogel 3D printing strategy based on ink with an ultra‐low storage modulus. By employing suspension printing, they achieved the precise fabrication of complex three‐dimensional structures while maximizing the retention of the aerogel's intrinsic porous characteristics without the need for additional rheological modifiers, thereby providing new insights for the design of highly flexible aerogel devices. Furthermore, Chen et al. [217] utilized 3D direct‐writing printing technology to fabricate SnO_2_ quantum dot/rGO composite aerogel sensors, achieving the controlled fabrication and mass production of complex structures. The resulting devices demonstrated a detection limit for formaldehyde as low as 8.02 ppb, with a power consumption of only approximately 130 µW. The above studies demonstrate that advancements in advanced manufacturing technologies are providing crucial support for the transition of gas‐sensitive aerogels from laboratory research to engineering applications (Figure 15d).

Overall, the high specific surface area, optimized pore structure, and heterojunction interfaces correspond to the adsorption and enrichment, diffusion and mass transfer, and signal conversion stages of the gas sensing process, respectively, and collectively determine the sensing performance of aerogels, meanwhile, long‐term stability, humidity tolerance, and scalability in manufacturing determine their potential for engineering applications. In the future, quantitative structure‐property relationships should be further established and combined with advanced manufacturing and interface control strategies to facilitate the transition of aerogel gas sensors from empirical optimization to rational design.

## 5 Conclusions and Outlook

Aerogels, characterized by their ultrahigh porosity, large specific surface area, and three‐dimensional interconnected network structure, provide a unique platform for the development of high‐performance gas‐sensing materials. In recent years, significant advances have been achieved in aerogel‐based gas sensors through engineering strategies such as compositional hybridization, defect regulation, heterostructure construction, and advanced manufacturing. These developments have markedly improved key sensing metrics, including detection limit, response speed, selectivity, and environmental adaptability.

This paper systematically reviews aerogel gas sensing systems from the perspective of the synergistic regulation of gas adsorption, diffusion‐based mass transfer, and electron transport, and establishes a conceptual framework linking structure, properties, and performance. Research indicates that the core advantage of high‐performance gas‐sensitive aerogels does not stem from a single material composition or structural parameter, but rather from their unique three‐dimensional porous framework, which simultaneously optimizes adsorption, diffusion, and electron transport processes. Specifically, the high specific surface area provides abundant active sites, the interconnected pore structure facilitates rapid gas transport, and the heterojunction interfaces enhance carrier migration and signal amplification. These three elements correspond to the key stages in the formation of the gas‐sensitive response, respectively, and together constitute the fundamental framework for the structure–property–performance relationship of aerogels. They serve as the crucial structural foundation for achieving high‐sensitivity, high‐selectivity, and high‐stability gas detection, and also provide a theoretical basis for the transition of aerogel gas sensing materials from empirical optimization to rational design.

Although significant progress has been made in aerogel gas sensing materials, several key challenges remain before they can be put into practical use. First, there is currently no consensus on the quantitative relationship between key parameters—such as specific surface area, pore size distribution, and heterojunction structures—and sensing performance, material design therefore relies heavily on empirical optimization. Second, achieving synergistic optimization among high sensitivity, high selectivity, and long‐term stability remains a core scientific challenge limiting the practical application of aerogel sensors. For example, while high specific surface area and highly active interfaces are beneficial for enhancing response performance, they may also lead to issues such as increased humidity sensitivity and reduced structural stability. Furthermore, traditional fabrication processes still suffer from limitations such as high costs, long lead times, and insufficient batch consistency, which hinder the large‐scale manufacturing and device integration of aerogel sensors.

To address these challenges, future research should focus on establishing quantitative structure‐property relationships, improving environmental stability, and enabling low‐cost, large‐scale manufacturing. By integrating emerging techniques such as in situ characterization, high‐throughput computing, and machine learning to develop quantitative predictive models linking aerogel structural parameters to sensing performance, we can drive a shift in material design from an empirical approach to a theory‐guided one. At the same time, by constructing hydrophobic interfaces, designing multi‐level pore structures, and optimizing heterojunctions, it is possible to achieve synergistic improvements in sensitivity, selectivity, and environmental stability. Combined with advanced manufacturing technologies such as 3D printing and directed assembly, this will enable low‐cost, scalable production. As these key issues are gradually resolved, aerogel gas sensors are expected to achieve further breakthroughs, moving from laboratory research to practical applications.

## Author Contributions

**Man Yuan**: investigation, methodology. **Shoutian Qiu**: supervision, funding acquisition, formal analysis. **Linheng He**: writing – review and editing, formal analysis, investigation. **Wei Liu**: writing – review and editing, supervision, formal analysis, writing – original draft, conceptualization. **Hao Guo**: writing – review and editing, writing – original draft, data curation, investigation, visualization. **Sheng Cui**: funding acquisition, supervision, writing – review and editing. **Qinxin Wang**: visualization, formal analysis, investigation.

## Conflicts of Interest

The authors declare no conflicts of interest.

## Data Availability

The data that support the findings of this study are available from the corresponding author upon reasonable request.
